# Diffusivity of CO_2_ in H_2_O: A Review of Experimental Studies and Molecular Simulations in the Bulk and in Confinement

**DOI:** 10.1021/acs.jced.3c00778

**Published:** 2024-03-20

**Authors:** H. Mert Polat, Felipe M. Coelho, Thijs J. H. Vlugt, Luís Fernando Mercier Franco, Ioannis N. Tsimpanogiannis, Othonas A. Moultos

**Affiliations:** † Engineering Thermodynamics, Process & Energy Department, Faculty of Mechanical Engineering, Delft University of Technology, Leeghwaterstraat 39, 2628CB Delft, The Netherlands; ‡ Universidade Estadual de Campinas (UNICAMP), Faculdade de Engenharia Química, Avenida Albert Einstein 500, Campinas, CEP: 13083-852, Brazil; § Chemical Process & Energy Resources Institute (CPERI)/Centre for Research & Technology Hellas (CERTH), 57001 Thermi-Thessaloniki, Greece

## Abstract

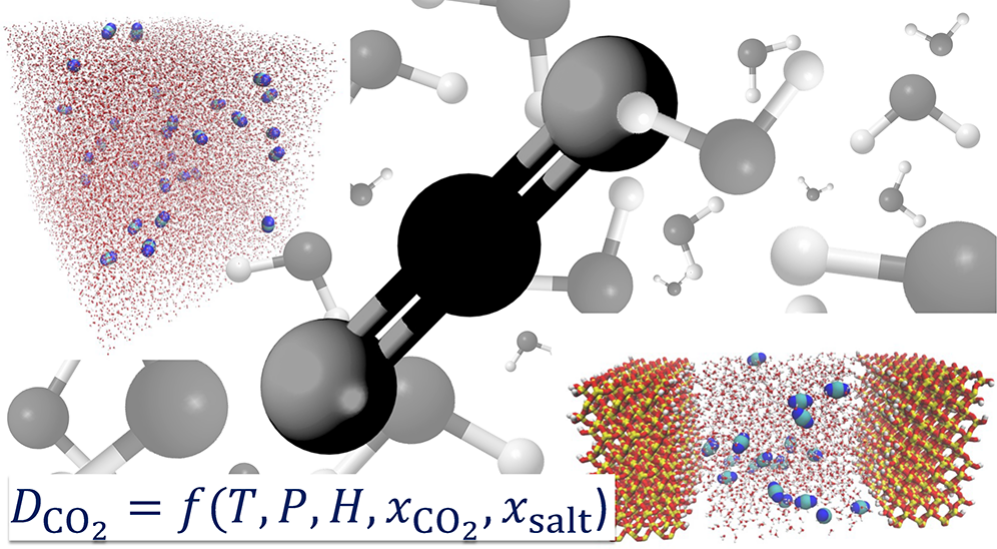

An in-depth review of the available experimental and molecular simulation studies of CO_2_ diffusion in H_2_O, which is a central property in important industrial and environmental processes, such as carbon capture and storage, enhanced oil recovery, and in the food industry is presented. The cases of both bulk and confined systems are covered. The experimental and molecular simulation data gathered are analyzed, and simple and computationally efficient correlations are devised. These correlations are applicable to conditions from 273 K and 0.1 MPa up to 473 K and 45 MPa. The available experimental data for diffusion coefficients of CO_2_ in brines are also collected, and their dependency on temperature, pressure, and salinity is examined in detail. Other engineering models and correlations reported in literature are also presented. The review of the simulation studies focuses on the force field combinations, the data for diffusivities at low and high pressures, finite-size effects, and the correlations developed based on the Molecular Dynamics data. Regarding the confined systems, we review the main methods to measure and compute the diffusivity of confined CO_2_ and discuss the main natural and artificial confining media (i.e., smectites, calcites, silica, MOFs, and carbon materials). Detailed discussion is provided regarding the driving force for diffusion of CO_2_ and H_2_O under confinement, and on the role of effects such as H_2_O adsorption on hydrophilic confining media on the diffusivity of CO_2_. Finally, an outlook of future research paths for advancing the field of CO_2_ diffusivity in H_2_O at the bulk phase and in confinement is laid out.

## 1 Introduction

The accurate knowledge of the intradiffusivity of CO_2_ in liquid H_2_O over a wide range of temperatures and pressures is crucial for the design and optimization of numerous industrial and environmental processes and applications. The most prominent applications are the following:

(i) *Carbon Capture& Sequestration (CCS).* CO_2_ is a greenhouse gas, produced from virtually every industrial process, and emitted into the atmosphere.^1−3^ In an effort to reduce the emissions of “man-made” CO_2_, and thus, partially mitigate the effects on the global climate change, CCS has been explored as a promising technology.^4^ CCS involves three major steps. At first, CO_2_ is captured from stationary CO_2_-intensive sources (i.e., fossil-fuel-burning power plants, cement, steel, hydrogen, ammonia, and other chemical industries).^5^ During the second step, the captured gas is transported through a network of pipelines to a permanent gas-storage site.^6,7^ During the third step, the captured gas is stored into subsurface, geological formations,^8−10^ such as active or depleted gas/oil reservoirs,^11−14^ saline aquifers,^15−22^ and methane-gas-producing coal deposits or unminable coal seams.^23−26^ The diffusivity of CO_2_ in aqueous solutions is an important transport property mainly encountered in steps one and three.
(ii) *CO*_*2*_*-based Enhanced Geothermal Systems (EGS).* Conventional geothermal systems that use H_2_O for the transmission of heat suffer from the drawback of fluid loss that has a significant negative economic effect. Dense-phase CO_2_ has thermal characteristics that allow it to transfer large quantities of heat, while at the same time having better physical characteristics (e.g., lower viscosity, higher compressibility, and expansibility).^27,28^ Therefore, CO_2_ has been considered for utilization in the process of geothermal energy by extracting heat from the ground.^29−31^ Such a process combines heat recovery from the subsurface, while the working fluid (e.g., CO_2_) losses can be considered as a part of CCS. In this way, value is added to the heat recovery process instead of considering it a financial loss, as occurs when using H_2_O as the working fluid. Depending on the depth from which heat is extracted, CO_2_ may encounter aqueous solutions, therefore CO_2_ dissolution, and subsequent diffusion in the aqueous phase, need to be studied to accurately describe the evolution of the CO_2_ plume. The flow of CO_2_ over aqueous brines is accompanied by a series of phenomena such as H_2_O evaporation^32,33^ and salt precipitation,^34,35^ thus affecting the porosity and permeability of the geologic formation.
(iii) *Enhanced Oil Recovery (EOR).* The injection of CO_2_ into an oil-producing reservoir has been considered as an alternative approach to increase oil production during tertiary oil recovery^36^ and is known as an EOR process. Usually EOR follows the secondary (i.e., waterflooding) oil recovery. Therefore, during the design of such a process, it is essential to account for the dissolution and diffusion of CO_2_ in the aqueous phase (e.g., either the formation water or the residual water after the waterflooding process).
(iv) *CO*_*2*_*ocean uptake.* Oceanic waters have absorbed approximately 40% of CO_2_ emissions since the beginning of the industrial era^37,38^ making the oceans the largest sink for anthropogenic CO_2_.^39^ Therefore, it is essential to accurately know the dissolution and diffusion mechanisms/parameters to delineate the amount of CO_2_ stored in the oceanic waters and its fate.
(v) CO_2_*in the food industry.* The diffusivity of CO_2_ in carbonated hydroalcoholic drinks, particularly in champagne, plays a pivotal role in influencing bubble dynamics and gas discharge kinetics, ultimately shaping the taste and mouthfeel of these beverages.^40^ Thus, the accurate knowledge of various thermophysical properties (with transport mechanisms being central) of CO_2_ in aqueous solutions relevant to this industry is essential for the production and quality control phases.

**Figure 1:**
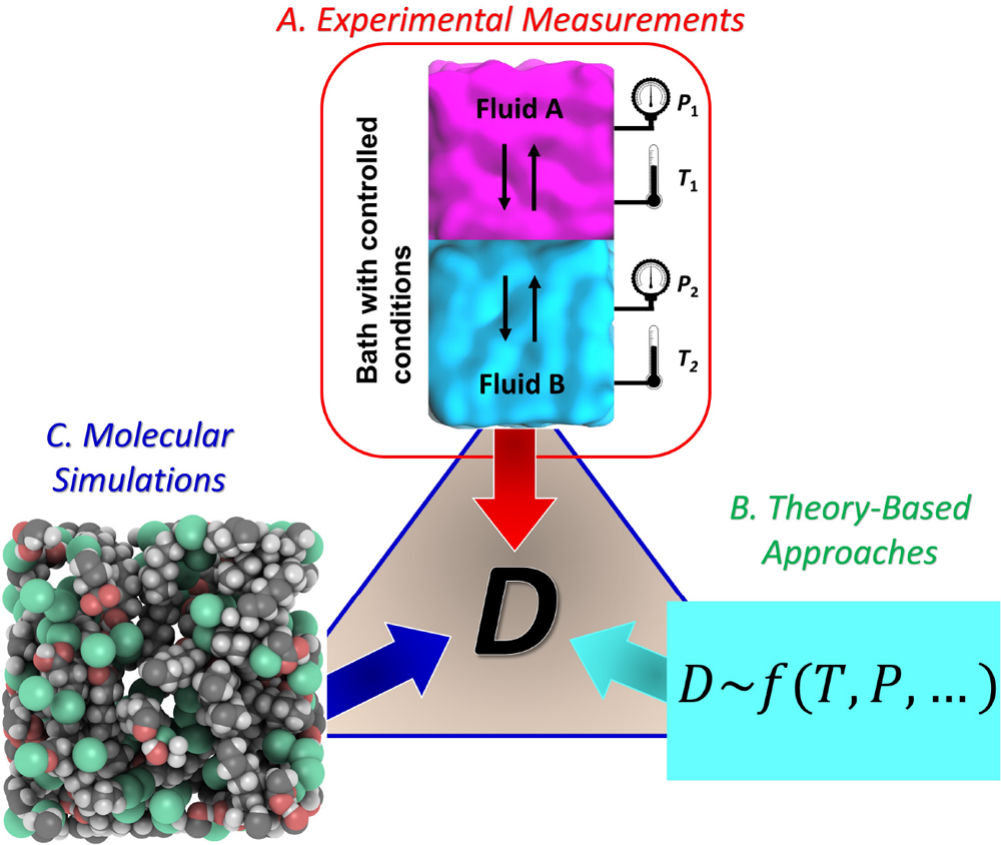
Schematic showing the common approaches considered for the estimation of diffusivities. A. Experiments, B. Theory-Based modeling, and C. Molecular Simulation.

Therefore, it becomes apparent that during the preliminary study, and the design and optimization of the processes described above, the accurate knowledge of the diffusivity of CO_2_ in liquid H_2_O under bulk conditions (applications (iv) and (v)) and in confined media (applications (i), (ii), and (iii)) is crucial. As shown in the schematic of Figure 1, the three major routes that are usually followed for the measurement/estimation of diffusion coefficients are experimental measurements, theoretical/semiempirical models, and molecular simulations, with the most common method used being molecular dynamics (MD).

At relatively low pressures (e.g., below 1 MPa), the solubilities of CO_2_ in H_2_O are rather low.^41^ For example, the solubility of CO_2_ in H_2_O at atmospheric pressure and temperatures in the range 303.15–363.15 K ranges from 5.03 × 10^–3^ to 6.50 × 10^–5^ (in mole fractions).^41^ At pressures up to 10 MPa and temperatures up to 423 K, the solubilities can increase by 2 orders of magnitude. For high pressures (i.e., 100 MPa), the solubilities can increase to a maximum of approximately 4.3 × 10^–2^. An extensive discussion on the effect of pressure on the solubility of CO_2_ in H_2_O can be found in a number of studies.^41−44^ Therefore, the intradiffusivity of CO_2_ in H_2_O essentially corresponds to the infinite dilution limit,^45^ since for most applications relatively low pressures and temperatures are concerned.

At higher pressures, at which the solubilities of CO_2_ in H_2_O are significantly higher than in the infinite dilution limit, it is of practical interest to measure/compute the mutual diffusivities (Fick and Maxwell-Stefan^46−49^) since the mass transport occurs due to gradients in chemical potentials.^46,50,51^ To this end, one can either use models that are based on the Darken equation^46,52,53^ or can follow the well-established methodology of computing the Maxwell-Stefan diffusivities (*Đ*_MS_) from the Onsanger coefficients in MD simulations,^47,49,54^ and the thermodynamic factor (Γ), e.g., from Kirkwood-Buff integrals^55−58^ or with Continuous Fractional Component Monte Carlo (CFCMC) simulations.^59^ In binary systems, Fick diffusivities follow from *D*_Fick_ = *ΓĐ*_MS_.^46−49^ In this review paper, we limit our attention to the diffusivity of infinite diluted CO_2_ in H_2_O. In this case, the intra-, Maxwell-Stefan, and Fick diffusivities are all equal *D*_self_ = *Đ*_MS_ = *D*_Fick_.^46^

Experimentally measured diffusivities are often scarcely available, and in most cases at/or close to the atmospheric pressure.^60,61^ A detailed discussion on how to overcome this lack of data through the use of semiempirical approaches is provided elsewhere.^50,53^ Namely, semiempirical correlations have been extensively used for obtaining the self- and intradiffusivity values at conditions outside the range of experimental measurements.^46,50,53,62,63^ The accuracy of such semiempirical methods depends on the extent and quality of the experimental measurements that have been used for their development and calibration. Although these methods are relatively easy to use and computationally fast, almost no insight into the physical mechanisms controlling the mass transport in the real system can be obtained.

Alternatively, approaches such as MD simulations can provide detailed physical insight;^64,65^ the downside being that they are significantly more computationally demanding compared to engineering models. During the past three decades years, MD has become a reliable and widely used approach for obtaining diffusivities of pure components and mixtures.^21,49,66−82^ This development is the direct result of a number of factors including: (i) the increase of available computational power, (ii) the availability and wide use of optimized open-source software,^83,84^ and (iii) the development of accurate force fields.^85−88^ The data obtained from MD simulations can be further used to devise engineering models and validate the semiempirical approaches.^72,89,90^ Macro-scale modeling approaches involving an equation-of-state such as PC-SAFT coupled with Stokes–Einstein equation or entropy scaling to compute self-diffusivities have been reported in literature, although, to the best of our knowledge, such methods have not been used to compute diffusivity of CO_2_ in H_2_O.^91−96^

This review paper focuses on: (a) reporting diffusivity data (experimental or from MD) of CO_2_ in pure H_2_O or brines, in bulk or under confinement, (b) providing engineering-type correlations of the collected data when possible, (c) critically discussing the insights from the literature, and (d) providing a few opinions to guide future developments. The remainder of this review paper is organized as follows: in Section 2, we examine the CO_2_ diffusion in bulk H_2_O, considering both experimental and MD studies. In Section 3 the corresponding cases under confinement are discussed. Finally, in Section 4 the future outlook and the conclusions are presented, respectively.

## 2 Aqueous CO_2_ Diffusion in the Bulk

### 2.1 Experimental Studies

#### 2.1.1 Experimental Measurement Techniques

Many different methods have been reported in the literature for the experimental measurement of the diffusion coefficients of gases in liquids, and have been extensively reviewed in a number of studies.^50,60,97−99^ Providing a detailed description of all these methods is beyond the scope of the current study. Instead, we provide a brief description of the experimental methods that have been used for the measurement of gas diffusivity in liquids, focusing primarily on those used for CO_2_ diffusing in H_2_O or brines. Such experimental methodologies include the following: (1) diaphragm cells,^100−102^ (2) wetted surface absorbers,^103,104^ (3) laminar jets,^105−108^ (4) capillary cells,^109,110^ (5) Taylor dispersion,^111−113^ (6) laser-induced fluorescence,^114^ (7) dynamic light scattering (DLS),^115^ (8) in situ Raman spectroscopy,^116,117^ (9) nuclear magnetic resonance (NMR) spectroscopy using pulsed field gradients (PFG),^118−120^ (10) pH-based methods,^121^ (11) pressure decay methods,^122−124^ and (12) dynamic interfacial tension method.^125^ Additional methodologies used for measuring gas diffusion in other liquids (e.g., CO_2_ in heavy oil or bitumen) include, but are not limited to, the following: (1) the dynamic pendant drop volume analysis (DPDVA),^126^ (2) the dynamic pendant drop surface analysis (DPDSA),^127^ (3) X-ray computer-assisted tomography (CAT) scanning,^128,129^ and (4) magnetic resonance imaging (MRI).^130^

**Figure 2:**
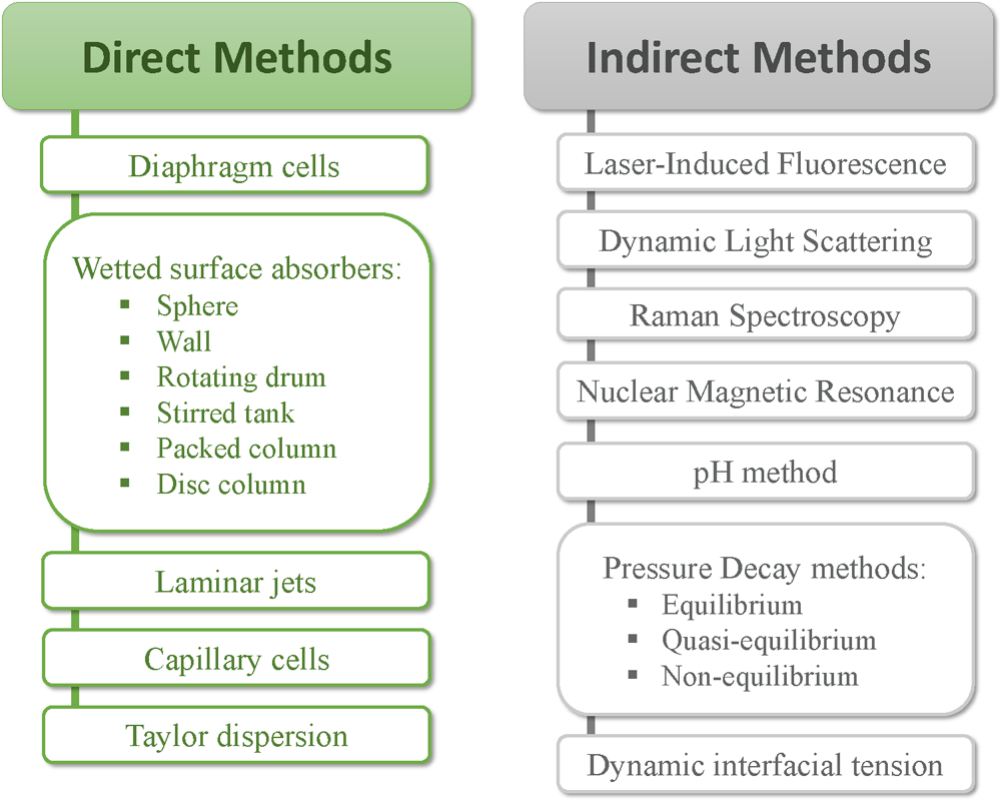
Direct and indicrect experimental methods that have been used for the measurement of gas diffusivity in liquids.

The experimental methods mentioned above can be divided into conventional (direct) and nonconventional (indirect),^99^ as shown in Figure 2. For the direct methods (e.g., diaphragm cells, wetted surface absorbers, laminar jets, capillary cells), it is essential to perform compositional measurements of fluid mixtures collected during the diffusion experiment to determine the gas diffusion coefficients. Therefore, direct methods are intrusive, can disturb the experiment if the removed samples are not minimal, can be time-consuming and labor intensive, are often expensive, and complex. These drawbacks are more pronounced when diffusion coefficients at higher temperatures/pressures are required.^122^ On the contrary, the indirect methods (e.g., laser-induced fluorescence, dynamic light scattering, Raman spectroscopy, nuclear magnetic resonance, pH-based methods, pressure decay methods) require less time compared to the conventional methods, and thus, are preferable in engineering applications.^123^ In these methods, the diffusion coefficients are indirectly determined by measuring a different property (e.g., interfacial tension, pH, gas pressure, gas volume, gas/liquid interface position) of the gas/liquid system that is known to be directly affected by the diffusion process. Diffusivity measurement methods, such as DLS, Raman spectroscopy, and NMR, that were mentioned earlier, or similar ones such as magnetic resonance imaging (MRI) and X-ray computer-assisted tomography (CAT) scanning (reported for the study of gas diffusion in hydrocarbon systems), require very expensive and highly sophisticated equipment, whose operation is limited to highly specialized technicians.^126^

In a diaphragm cell, two solutions of different composition are brought into contact by a diaphragm. The cell usually has to be calibrated with a mixture of known diffusion coefficient. The method has a significant drawback, since it requires a large amount of time (i.e., 2–3 days) for each measurement.

In the wetted surface absorber technique, absorption takes place in a thin laminar film flowing over a surface of defined geometry, such as a sphere^104^ or a wall/plate.^131,132^ This method has the following two limitations that are important only at high degrees of liquid saturation: (i) the finite thickness of the liquid film, which absorbs finite amount of gas, and (ii) nonuniform velocity profile. Olbrich and Wild^133^ extended the earlier mathematical analysis of Davidson^134^ for absorption on a sphere to any flow geometry exhibiting a certain degree of symmetry. In a similar manner, the laminar jet method is based on the gas absorption taking place in a free-flowing laminar jet. Both these methods require knowledge of the fluid dynamics for the analysis and calculation of the diffusion coefficients. Tang and Himmelblau^131^ reviewed other gas–liquid contacting devices that have been used in fundamental studies including the rotating drum,^135^ the stirred tank,^136^ the packed column, and the disk column,^137^ and concluded that in such devices it is difficult to measure the hydrodynamic characteristics of the liquid phase, therefore making it difficult to interpret the obtained diffusivity results.

Single^110^ or multiple^109^ capillary cells (with the capillaries having size of approximately 1 mm) are used to restrict liquid convection within the capillaries. The liquid component is placed in the capillary and then brought into contact with the second diffusing component. If diffusion is allowed to proceed until the steady-state is reached, then the rate of diffusion can be described by relatively simple mathematics. This method has, however, two disadvantages: (i) solubility data are required; therefore, the accuracy of diffusivity depends on the accuracy of the solubility data, and (ii) as a result of the gas absorption rate being measured volumetrically, accurate diffusivities are limited only to systems for which gas solubilities are at least moderate.

The measurement of the diffusion coefficient of a gas in a solvent with the Taylor dispersion technique requires the simultaneous injection of a sample of a solution containing the gas and the solvent into a stream of the pure solvent while the dispersion of the gas during the laminar flow through a capillary is monitored. In this approach, the parabolic flow profile results in spreading the solute pulse out longtitudinally, while simultaneously radial diffusion acts to keep the pulse confined. Extracting the diffusion coefficient from the mathematical analysis of this problem is based on the seminal work of Taylor^111^ and Aris.^112^ An extensive discussion on the accuracy of this methodology has been presented by Alizadeh et al.^138^ For experimental studies using the Taylor dispersion method for the diffusivity of CO_2_ in H_2_O, the reader is referred to refs (113, 139−141).

By performing an analysis of the intensity of the quasielastically scattered light, a number of thermophysical properties (i.e., viscosity, surface tension, speed of sound, thermal diffusivity) can be determined in an absolute way by using dynamic light scattering (DLS).^115^ Klein et al.^142^ provided a comprehensive description of the techniques used, including the optical and electronic arrangement of the setup used for performing those measurements. Therefore, when applying DLS to the bulk of fluids which are at macroscopic thermodynamic equilibrium, the mean lifetimes of fluctuations in concentration, temperature or entropy, and pressure are analyzed by calculating the correlation function (CF) of the scattered light intensity. By such an analysis the thermophysical properties of interest can be extracted. Contrary, inelastically scattered light analyzed by Raman spectroscopy can provide insight into the molecular structure. In situ Raman spectroscopy in horizontal fused silica capillary has become a powerful technique utilized to determine CO_2_ diffusion coefficients at high pressures and temperatures.^116,117^ In an alternative approach, Hirai et al.^114^ used laser-induced fluorescence (LIF) to measure CO_2_ dissolution in water under high pressures.

The PFG-NMR methodology is a noninvasive means for measuring translational motion and is based on the use of magnetic gradient fields which imprint phase shifts on the nuclear spins of the diffusing species.^120^ For cases in which an increase in gradient strength or the Brownian motion is present, a decrease in NMR signals is observed. As a result, the molecular motion can be quantified and the self-diffusion coefficient can be obtained. The PFG-NMR method does not require any calibration or additional information on the investigated systems, which constitutes an advantage of this method when compared to others discussed earlier. A detailed description of the theory behind this method, as well as the experimental aspects associated with the method, can be found in the review articles of Price.^143,144^

Sell et al.^121^ utilized a microfluidic-based approach to measure the mutual diffusion coefficient of carbon dioxide in water and brine. With their approach the diffusion is quantified by imaging fluorescence quenching of a pH-dependent dye, and subsequent mathematical analysis. An important advantage of the method is the efficacy and speed of the diffusivity measurements. The authors reported measurements completed in less than 90 s, which should be compared to hours or days required by other methods.

The pressure decay method is considered the most widely applied indirect method for the measurement of gas diffusivities in the liquid phase.^99,123^ The method was established by Riazi^145^ for the measurement of diffusion coefficients of gas in hydrocarbon systems. The method is based on the measurement of the decrease in the pressure of gas in direct contact with a liquid at a constant temperature *PVT* setup or diffusion cell. To obtain the gas diffusion coefficient, the pressure decay data as a function of time are matched with a mathematical model. Therefore, such an approach makes the calculation of the diffusion coefficient dependent on how detailed the mathematical model used for the analysis is.^146^ This issue becomes more evident when the pressure decay method is used to measure the diffusivity of CO_2_ in H_2_O where the density-driven convection needs to be considered.^147,148^ A number of studies^124,147,149−154^ used the pressure decay method for the measurement of the diffusivity of CO_2_ in H_2_O.

Based on the boundary condition of the gas/liquid interface used in the modeling of the pressure decay method, Tharanivasan et al.^155^ recommended the classification of the mathematical models under three categories as follows: (i) equilibrium, (ii) quasi-equilibrium, and (iii) nonequilibrium. The first category considers that the concentration on gas/liquid interface is constant and always equal to the equilibrium concentration. An important limitation of the models belonging to the first category is that the decay in pressure of the gas phase should be very small; otherwise, higher errors (originating from the assumption of constant equilibrium concentration at the interface) will occur when the model is used to analyze pressure-decay data. Models of the second category consider a nonconstant concentration, corresponding to the existing cell pressure at the gas/liquid interface, resolving thus, the deficiency of the equilibrium model. However, for quasi-equilibrium models an exact analytical solution has not been reported to date. Finally, the nonequilibrium models^156,157^ assume that a mass transfer resistance is considered at the gas/liquid interface. Such an assumption, however, is still under scientific debate.^123^

The dynamic interfacial tension method^125^ is capable of simultaneously determining the gas diffusion coefficient and the interface mass transfer coefficient in a liquid. Initially, the dynamic and equilibrium interfacial tensions of the gas–liquid system are measured by using the axisymmetric drop shape analysis (ADSA) technique for the pendant drop case. Next, a mathematical model is developed to study the mass transfer in the gas–liquid system. The gas diffusion coefficient in the liquid is used as an adjustable parameter and is the result of an optimization process to match the numerically calculated and experimentally measured dynamic interfacial tensions.

#### 2.1.2 Correlation of Experimental Data

Mutoru et al.^61^ presented a comprehensive collection of experimental data of CO_2_ diffusion in bulk pure H_2_O that are available in the open literature. This database covers studies up to 2010, and includes 150 experimental data points (also incorporating the experimental data from the earlier review by Himmelblau^60^), the majority of which are at pressure equal to 0.1 MPa. Mutoru et al.^61^ presented a detailed discussion of mean-field-theory models that consider the diffusion coefficient of CO_2_ in H_2_O. They also reported a novel methodology for the calculation of the diffusion coefficient at infinite dilution of either of the two components. Magalhães et al.^158^ examined the performance of a number of empirical correlations for the diffusion coefficients of CO_2_ in H_2_O. The experimental data were correlated as a function of temperature and the viscosity or density of the solvent. For the particular system they limited their study to 111 experimental data points that are mainly at 0.1 MPa (all data were included in the database of Mutoru et al.^61^).

Since the methodology of Mutoru et al.^61^ seems to be in principle accurate, and general in nature, it can be used for computing the diffusion coefficient of other gases in H_2_O as well, but requires significant computational effort to be applied. This section is motivated by the need to develop an equally accurate method for the calculation of the diffusion coefficient of CO_2_ in H_2_O, yet simple enough to be used in reservoir simulators, where the repeated use of the diffusivity correlation is required. In reservoir simulators,^159^ the domain of interest is discretized in a (usually) large number of grid-blocks, and the balance equations of momentum, mass, and energy need to be numerically solved in each one of them, while the solution process is repeated for all the time-steps considered.^160^ To this purpose, two different groups of correlations are examined. The first considers two Arrhenius-type correlations,^116,161^ while the second group considers the Speedy-Angel power-law type of correlation.^162^

Two are the major advantages of the correlations that were examined in the current study: (i) they are equally accurate at low pressures (0.1 MPa) and provide higher accuracy at pressures that are higher than atmospheric, and (ii) they are simple to use, and therefore, they are computationally efficient, and thus can be used during the process design and optimization. However, they are component-specific, therefore they are not general in nature. To examine different diffusion systems, the parameters of the equations need to be refitted to the corresponding, component-specific experimental diffusivity data.

Initially, we briefly present three correlations that have been reported in literature. Next, the three correlations are fitted to the experimental data used for the development of the Mutoru et al.^61^ methodology to obtain the correlation parameters. Then, the three correlations, and the methodology of Mutoru et al.^61^ are extrapolated to pressure and temperature conditions that are outside the range of development, and are compared to the experimental data of Lu et al.^116^ and Cadogan et al.^113^ Finally, an extended experimental database that includes the database of Mutoru et al.^61^ and the experimental studies of Lu et al. and Cadogan et al. is used to re-evaluate the parameters for the three correlations. The new correlations are further tested against experimental data at higher pressures that have not been included in the correlation development.

#### 2.1.3 Model Development for Diffusion in Pure H_2_O

Here, we consider three literature-reported correlations presented in eqs 1–3 below to describe the experimental data of the diffusion coefficient of CO_2_ in pure H_2_O collected by Mutoru et al.^61^ This database is termed “original” in Table 1. The term “limited” in the same table corresponds to the experimental data used by Versteeg et al.,^161^ which is a subset of the “original” database that contains only 30 experimental data points. “new data” correspond to the experimental values reported by Lu et al.,^116^ and Cadogan et al.^113^ that are at higher pressures. The three correlations examined have been previously reported in the literature, and have been used in a number of studies to correlate experimental data^116,161^ or molecular simulation results.^45,163^

**Table 1:** Percentage Average Absolute Deviation (% AAD) between Experimental Data and Correlations for the Diffusion Coefficient of CO_2_ in H_2_O^a^

case	parameter fitting	data base used for %AAD calculations	ARR (%AAD)	VTF (%AAD)	SA (%AAD)	Mutoru et al. (%AAD)
1	original	original	4.9	5.0	5.7	4.9
2	original	new data	24.6	30.8	16.4	11.5
3	extended	extended	11.2	5.7	6.9	na
4	extended	new data	10.8	7.7	7.5	na
5	limited	limited	3.7	3.7	4.3	3.7
6	limited	original	5.1	5.2	5.2	na
7	limited	extended	7.4	7.4	5.9	na
8	limited	new data	17.2	17.2	8.7	na

a Notation for the experimental data: Original: database reported by Mutoru et al.;^61^ New data: Lu et al.,^116^ Cadogan et al.;^113^ Extended: Original + New data; Limited: database reported by Versteeg et al.;^161^ na: not applicable. ARR stands for the Arrhenius equation (eq 1), VTF stands for the Vogel–Tamann–Fulcher equation ( eq 2), and SA stands for the Speedy-Angell power-law equation ( eq 3).

Versteeg et al.^161^ used a limited number of experiments (i.e., 30 data points of diffusion coefficients of CO_2_ in H_2_O at 0.1 MPa and for temperatures up to 348 K) and fitted the experimental data to an Arrhenius-type equation (denoted with superscript “ARR”) given as follows:
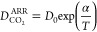
1where *D*_0_, and α are fitting parameters, and *T* is the temperature. The correlation has high accuracy within the range of development (i.e., for temperatures up to 348 K). Moultos et al.^45^ showed that the extrapolation of the correlation by Versteeg et al.^161^ to temperatures higher than 348 K deviates significantly from recent experimental data that were not included in the original development of the Arrhenius-type correlation.

This is clearly shown in Table 1 where the percentage average absolute deviation (%AAD), defined as , is given for a number of different cases. The superscripts “calc” and “exp” denote the computed and experimental values respectively of the diffusion coefficients, *D*_CO_2__, of CO_2_ in H_2_O. When the correlation by Versteeg et al.^161^ is used to compute the *D*_CO_2__ at the temperatures in the “limited” database, it produces a value for %AAD equal to 3.7% (i.e., case 5). Alternatively, %AAD rises to 17.2% when the experimental data of Lu et al.^116^ and Cadogan et al.^113^ are considered (i.e., case 8).

Lu and co-workers^116^ used a modified Arrhenius-type of equation, known as the Vogel–Tamann–Fulcher (denoted with superscript “VTF”) to correlate the experimental data from a new set of experimental measurements that they performed in the pressure range 10–45 MPa and temperature range 268–473 K. The VTF equation is given as
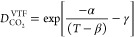
2where α, β, and γ are fitting parameters. Lu et al.^116^ found better agreement, however, with their experimental data when they used a power-law-type of equation expressed as follows:
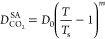
3where *D*_0_, *T*_s_, and *m* are fitting parameters. In most cases, *T*_s_ = 227 K. This type of correlation is known as the Speedy-Angell power-law equation^162^ and is denoted with the superscript “SA”.

#### 2.1.4 Results for Experimental Data at Low Pressures

Initially, we used the experimental data collected by Mutoru et al.^61^ to perform comparisons between the methodology of Mutoru et al.^61^ and the three correlations examined here. The computed values for the parameters of the three correlations are reported in Table 2.

**Table 2:** Parameters for the Diffusion Coefficient of CO_2_ in H_2_O Calculated Using Different Correlations^a^

correlation	*D*_0_ (m^2^ s^–1^)	*m*	α	β	γ
ARR-type	3.657 × 10^–6^	na	2.2546 × 10^3^	na	na
VTF-type	na	na	4.3152 × 10^3^	–123.2149	9.84018
SA-type	19.798 × 10^–9^	2.01489	na	na	na

a The case of using the “Original” database.

The values for the %AAD in calculating the diffusion coefficients of CO_2_ in H_2_O for the three correlations are given in Table 1. All correlations are in very good agreement with the methodology of Mutoru et al.^61^ with the Arrhenius-type (ARR-type) correlation having the lowest %AAD. When the pressure is equal to 0.1 MPa, the temperature range of applicability of the three correlations, as well as the methodology of Mutoru et al.,^61^ is limited to temperatures up to 373 K.

**Figure 3:**
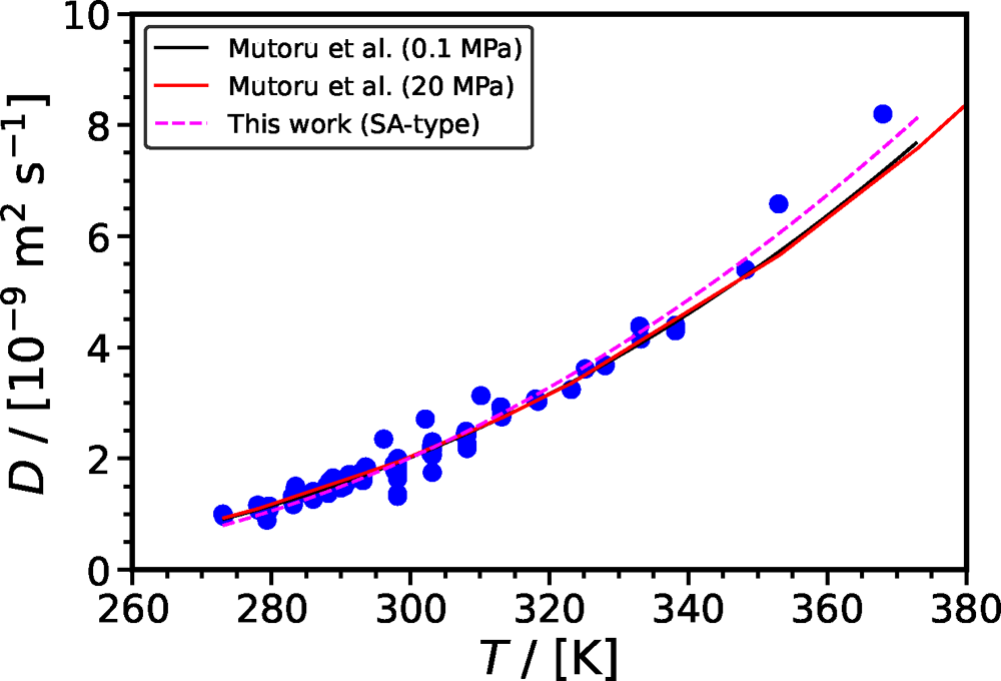
Diffusion coefficient of CO_2_ in H_2_O as a function of temperature. Circles denote the experimental data collected by Mutoru et al.^61^ The solid lines denote the calculations using the methodology by Mutoru et al. [calculations at: 0.1 MPa (black line), and 20 MPa (red line)]. The magenta dashed line denotes the correlation (SA-type) developed in this work.

The good agreement between the experimental data and the methods considered is also demonstrated in Figure 3, where *D*_CO_2__ is shown as a function of temperature. In Figure 3, we show only the SA-type correlation, which performs the least satisfactory among the three correlations considered. Yet we can observe that this correlation follows very closely the calculations using the methodology of Mutoru et al.^61^

The calculations discussed so far correspond to pressures that are equal to 0.1 MPa. Figure 3 also shows the calculations of *D*_CO_2__ using the methodology of Mutoru et al.,^61^ however at pressure equal to 20 MPa. The resulting curve for the diffusion coefficient of CO_2_ in H_2_O as a function of temperature is practically indistinguishable from the case of 0.1 MPa. Pressure effects on the diffusion coefficient of CO_2_ in H_2_O is addressed further in the following section.

#### 2.1.5 Results for Experimental Data at High Pressures

The extensive experimental studies by Lu et al.^116^ and Cadogan et al.^113^ have shown that pressure has a very limited effect on the diffusion coefficient of CO_2_ in H_2_O, up to 45 MPa and temperatures up to 473 K. This is expected due to the low compressibility of liquid H_2_O at these conditions. For this pressure and temperature range, a similar conclusion was reached from the MD simulations reported by Moultos et al.^45^ Interestingly, MD simulations show that pressure effects could become significant at higher temperatures and pressures.

**Figure 4:**
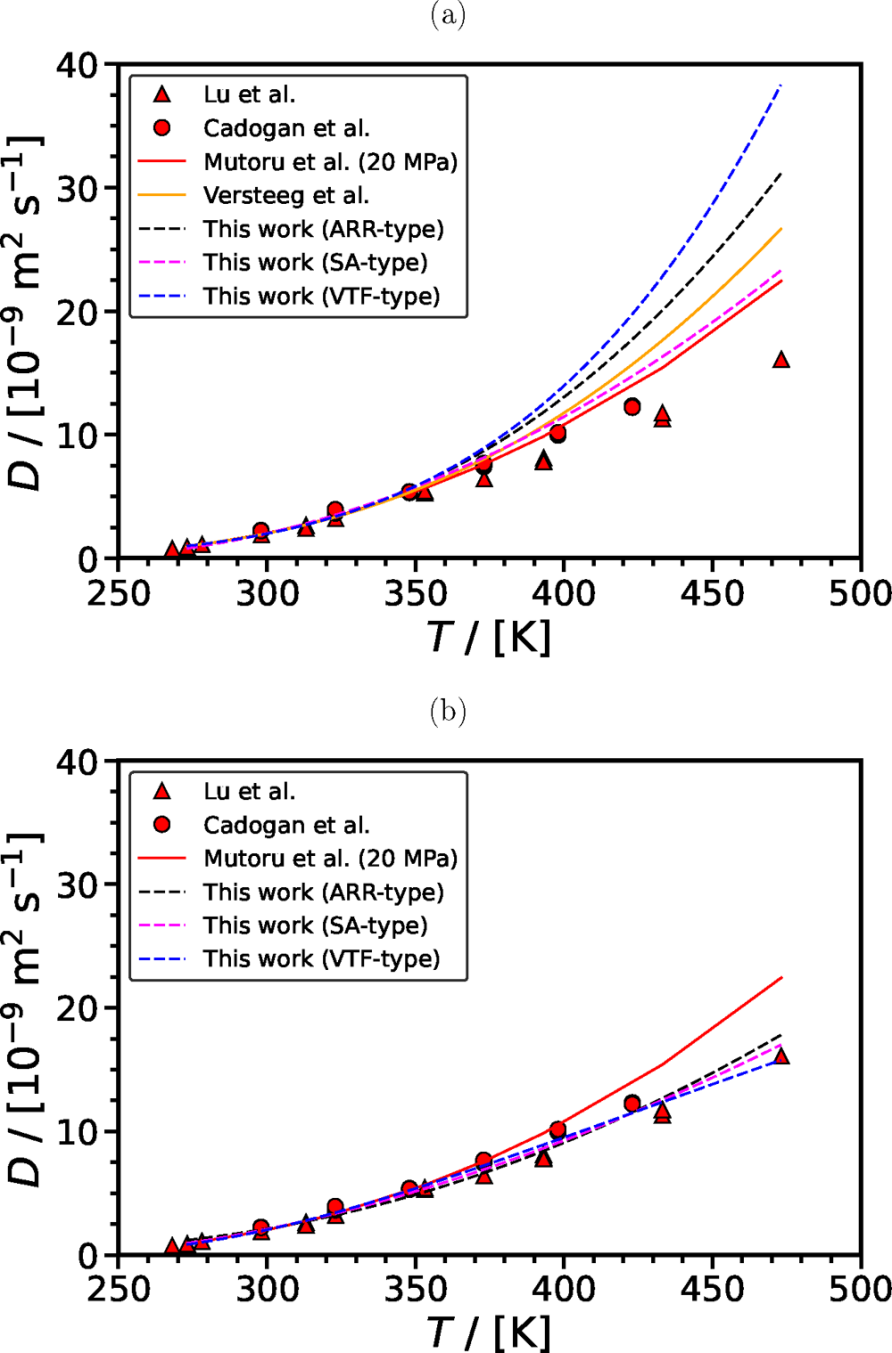
Diffusion coefficient of CO_2_ in H_2_O as a function of temperature. Triangles denote the experimental data by Lu et al.,^116^ and circles the experimental data by Cadogan et al.^113^ The red solid line denotes the calculations of the diffusion coefficient of CO_2_ in H_2_O at various temperatures and 20 MPa using the correlation of Mutoru et al.,^61^ and the orange solid line denotes the calculation using the correlation of Versteeg et al.^161^ The dashed lines denote the correlations examined in this work (SA-type: magenta; VTF-type: blue; ARR-type: black): (a) Extrapolation and comparison with recent experimental data at higher pressures. (b) The parameters for the three correlations are re-evaluated to include recent experimental data (Lu et al.,^116^ Cadogan et al.^113^).

Therefore, for all practical engineering applications at the conditions where pressure has a negligible effect on the diffusion coefficient of CO_2_ in H_2_O, one could use the correlation that gives the diffusion coefficient of CO_2_ in H_2_O only as a function of temperature (which is independent of pressure). Essentially, one could use the correlations developed in section 2.1.2 (i.e., for pressures equal to 0.1 MPa and temperatures up to 373 K) or the methodology developed by Mutoru et al.^61^ In Figure 4(a), the diffusion coefficient of CO_2_ in H_2_O is shown as a function of temperature for temperatures up to 473 K. Namely, we extrapolate the use of the methodology of Mutoru et al.^61^ or the three correlations by 100 K. These calculations are compared with the experimental data by Lu et al.,^116^ and Cadogan et al.,^113^ which are at higher pressures. While very good agreement is observed for temperatures lower than 373 K, deviations increase significantly for higher temperatures as can be seen by the values of %AAD listed in Table 1. Among all cases considered in this section, the methodology of Mutoru et al.^61^ performs better in predicting the diffusion coefficient of CO_2_ in H_2_O under extrapolated conditions.

#### 2.1.6 Results for Combined Experimental Data

Motivated by the observations in the previous section, we re-evaluate the parameters of the three correlations using the “extended” database that includes also the experimental data of Lu et al.^116^ and Cadogan et al.^113^ at higher pressures and temperatures in addition to the experimental data collected by Mutoru et al.^61^ The new parameters that resulted from the fitting are reported in Table 3. %AAD in calculating *D*_CO_2__ for the three correlations are also given in Table 1. Significant improvements can be observed in the calculations of the diffusion coefficients of the experimental data of Lu et al.^116^ and Cadogan et al.^113^ In particular, for the case of the SA-type correlation, the %AAD drops from 16.4% to 7.5% when the new parameters are used. The improvement is more pronounced for the case of the VTF-type correlation. The %AAD drops from 30.8% to 7.7% when the new parameters are used. An intermediate behavior is observed for the case of the ARR-type correlation (the %AAD drops from 24.6% to 10.8%).

**Table 3:** Parameters for the Diffusion Coefficient of CO_2_ in H_2_O Calculated Using Different Correlations^a^

correlation	*D*_0_ (m^2^ s^–1^)	*m*	α	β	γ
ARR-type	0.7056 × 10^–6^	na	1.7407 × 10^3^	na	na
VTF-type	na	na	0.52369 × 10^3^	159.003	16.2975
SA-type	14.802 × 10^–9^	1.72362	na	na	na

a The case of using the “extended” database.

Figure 4(b) shows the diffusion coefficient of CO_2_ in H_2_O as a function of temperature. As can be seen, the reparameterized correlations are in very good agreement with the experimental values at higher temperatures and pressures. All three correlations examined here perform better than the methodology of Mutoru et al.^61^

**Figure 5:**
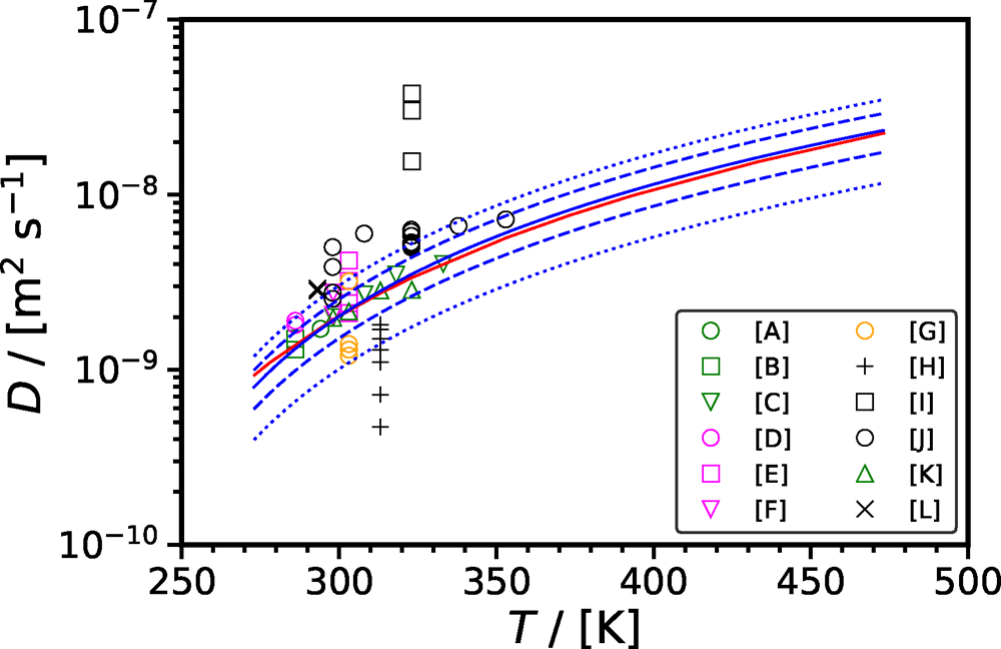
Diffusion coefficient of CO_2_ in H_2_O as a function of temperature. The solid red line denotes the calculations using the methodology by Mutoru et al. at 20 MPa. The solid blue line denotes the correlation (SA-type) developed in this work, while the dashed and dotted blue lines indicate the boundaries of ±25% and ±50% of the diffusion coefficient computed with the SA correlation, respectively. Symbols denote the experimental data. Legend: [A] Belgodere et al.;^117^ [B] Hirai et al.;^114^ [C] Bellaire et al.;^120^ [D] Shimizu et al.;^164^ [E] Tomita et al.;^165^ [F] Farajzadeh et al.;^147^ [G] Chiquet;^166^ [H] Tewes and Boury;^167^ [I] Li et al.;^168^ [J] Ahmadi et al.;^169^ [K] Lee et al.;^132^ and [L] Basilio et al.^124^

The proposed correlations are further tested with some additional experimental data at higher pressures which are indicated in Figure 5. These experimental studies have not been included in any of the databases^61,158,161^ discussed in the previous sections. The figure shows the comparison of the experimental diffusion coefficient of CO_2_ in H_2_O as a function of temperature against calculations using (i) the Mutoru et al. methodology (at 20 MPa) and (ii) the SA correlation developed in the current study. The dotted lines indicate the boundaries of ±25% and ±50% of the diffusion coefficient calculated with the SA correlation. There are four different groups of experimental data which are indicated by a different color in the figure. The experimental data (green symbols) of Belgodere et al.,^117^ Hirai et al.,^114^ Bellaire et al.,^120^ and Lee et al.^132^ are in good agreement with the SA correlation. Most of the experimental data (magenda symbols) of Shimizu et al.,^164^ Tomita et al.^165^ and Farajzadeh et al.^147^ fall in the zone of ±25% from the SA correlation (with a limited number of experimental data falling outside). The experimental data (orange symbols) of Chiquet^166^ fall in the range ± (25–50)% from the SA correlation. Finally, the experimental data (black symbols) of Tewes and Boury,^167^ Li et al.,^168^ Basilio et al.,^124^ and Ahmadi et al.^169^ exhibit deviations which can be significantly higher than 50%.

#### 2.1.7 Diffusion in Brines

Only a limited number of experimental measurements has been reported for the case of CO_2_ diffusing in brines of various compositions. Table 4 shows a number of experimental studies that were identified in this review. The table also shows the range of parameters examined and the different brines considered.

**Table 4:** List of Experimental Studies for the Diffusion Coefficient of CO_2_ in Aqueous Brines

year	authors	*T* range (K)	*P* range (MPa)	*D* range (10^–9^ m^2^ s^–1^)	salinity	variable	brine solution
1959	Nijsing et al.^173^	298.15	0.1	1.06–1.95	0–1.28 mol L^–1^	*S*	Na_2_SO_4_; MgSO_4_
1963	Ratcliff & Holdcroft^170^	298.15	0.1	1.28–1.84	0.32–3.78 mol L^–1^	*S*	NaCl; NaNO_3_; Na_2_SO_4_; MgCl_2_; Mg(NO_3_)_2_; MgSO_4_
1996	Wang et al.^174^	311.15	1.524–5.178	2.925–4.827	0.25 N	*P*	NaCl
2006	Yang & Gu^149^	300.15, 331.15	2.6–7.54	170.7–269.8	4310 mg L^–1^	*P*	reservoir (Instow) brine
2006	Yang et al.^125^	300.15	0.1–6	0.31–1.34	64 160 mg L^–1^	*P*	reservoir (Weyburn) brine
2008	Bahar & Liu^175^	356.15	na	na	2 wt %	na	NaCl
2013	Azin et al.^151^	305.15–323.15	5.9–6.9	3.52–6.16	182 513 mg L^–1^	*T*, *P*	Aquifer brine
2013	Wang et al.^176^	318.15	3.43–8.02	233.6–251.34	6778 mg L^–1^	*P*	reservoir brine
2015	Cadogan et al.^119^	298.15	0.1	1.25–2.13	0–5 mol kg^–1^	*S*	NaCl; CaCl_2_; Na_2_SO_4_
2015	Belgodere et al.^117^	294.15	4	0.93–1.71	0–6 mol kg^–1^	*S*	NaCl
2015	Zhang et al.^153^	298.15	1.17	1.5–1.91	0–100 000 ppm	*T*, *P*, *S*	NaCl; Na_2_SO_4_; NaHCO_3_; MgCl_2_; CaCl_2_
2015	Jafari et al.^152^	303.15, 313.15	5.459–6.10	0.678–23.3	0–200 000 mg L^–1^	*P*, *S*	NaCl; KCl; CaCl_2_; MgCl_2_; reservoir brine
2017	Zarghami et al.^177^	341.15	1.745	6.5–8.2	0–80 ppt	*T*, *S*	NaCl
2017	Shu et al.^178^	293.15	1.7–2.2	18.08–22.42	3 wt %	*P*	NaCl
2017	Shu et al.^179^	293.15, 303.15	1.77–2.22	1.0–3.5	3 wt %	*P*	NaCl
2018	Shi et al.^154^	323.15	4.25–5.786	1.25–293	248 991 mg L^–1^	na	reservoir (Mt. Simon) brine
2018	Perera et al.^172^	323.15	9	1.72–3.08	0–4 M	*S*	NaCl
2018	Li et al.^180^	313	2–8	1.92–2.1	1 M	*P*	NaCl + KI
2019	Tang et al.^181^	355.65	14–24	4.98–9.04	243 143 mg L^–1^	*P*	reservoir brine
2019	Tang et al.^181^	293–393	20.2	3.09–8.46	243 143 mg L^–1^	*T*	reservoir brine
2023	Zhang et al.^182^	286.15–303.15	0.1–5	0.126–0.73	3 wt %	*T*, *P*	NaCl
2024	Basilio et al.^124^	293.15	1.5	1.28–2.91	0–5 mol L^–1^	*S*	NaCl

**Figure 6:**
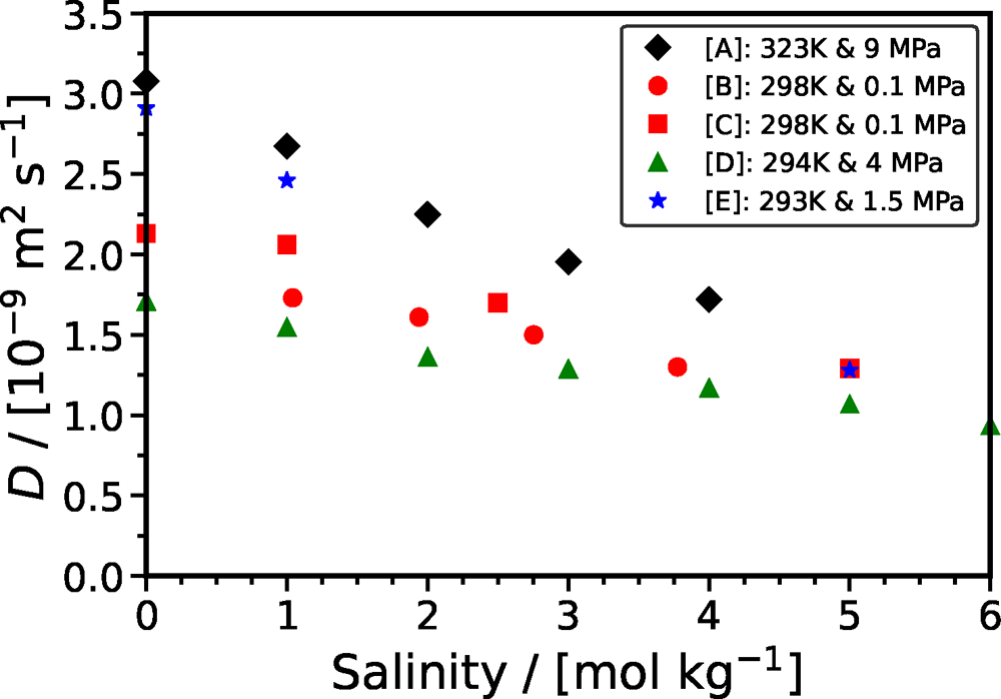
Effect of temperature and salinity [mol NaCl/(kg H_2_O)] on the diffusion coefficient of CO_2_ in brines. Symbols denote the experimental data. Legend: [A] Perera et al.;^172^ [B] Ratcliff and Holdcroft;^170^ [C] Cadogan et al.;^119^ [D] Belgodere et al.;^117^ and [E] Basilio et al.^124^

Figure 6 shows the effect of salinity [in units of mol NaCl/(kg H_2_O)] on the diffusion coefficient of CO_2_ in various aqueous solutions of NaCl. Each set of experiments is performed at constant temperature and pressure. We observe that for a constant temperature and pressure the diffusivity of CO_2_ in the brine decreases as the salinity increases. This observation is confirmed by all five experimental studies considered in Figure 6. As expected, higher temperatures result in higher diffusivities.

The experimental data of Ratcliff and Holdcroft^170^ and Cadogan et al.^119^ exhibit an increasing deviation at lower salinity values, even though both studies are performed at the same *P* and *T* conditions. In particular, for the case of salinity equal to 1 mol L^–1^, the CO_2_ diffusivity reported by Cadogan et al. (using the Taylor dispersion method) is higher than the value reported by Ratcliff and Holdcroft (using the wetted sphere absorber technique) by approximately 16%. For the limiting case of pure H_2_O, Cadogan et al. reported a diffusivity equal to (2.130 ± 0.028) × 10^–9^ m^2^ s^–1^, while the calculation with the method of Mutorou et al.^171^ resulted in a value equal to (1.927 ± 0.001) × 10^–9^ m^2^ s^–1^, while the calculation with the SA-type correlation of this study resulted in a value equal to (1.917 ± 0.001) × 10^–9^ m^2^ s^–1^. Both calculations indicate that the experimental measurements of Cadogan et al. seem to be overestimated.

Finally, the experimental data of Basilio et al.^124^ performed at 293 K and 1.5 MPa have higher values than the data from Belgodere et al.^117^ performed at 294 K and 4 MPa. A similar analysis indicates that the data of Basilio et al. are higher than expected when compared to calculations with the method of Mutorou et al. and the SA-type correlation. For example, the SA-type correlation estimates the diffusivity to be (1.664 ± 0.001) × 10^–9^ m^2^ s^–1^ and (1.715 ± 0.001) × 10^–9^ m^2^ s^–1^ for 293 and 294 K, respectively. However, Basilio et al. (at 293 K) reported an experimental value equal to 2.91 × 10^–9^ m^2^ s^–1^, while Belgodere et al. (at 294 K) reported an experimental value equal to 1.71 × 10^–9^ m^2^ s^–1^.

**Figure 7:**
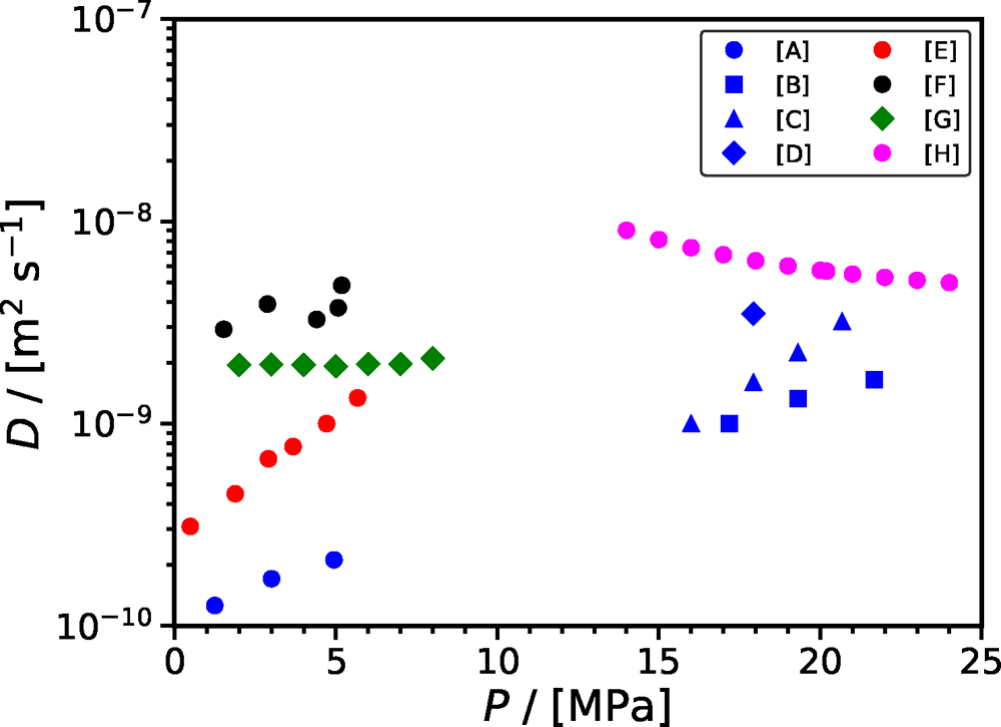
Effect of pressure and salinity on the diffusion coefficient of CO_2_ in aqueous brines as a function of pressure. Symbols denote the experimental data. Legend: [A] Zhang et al.^182^ (288.15 K & 3 wt % salinity); [B] Shu et al.^178^ (293.15 K & 3 wt % salinity); [C] Shu et al.^179^ (293.15 K & 3 wt % salinity); [D] Shu et al.^179^ (313.15 K & 3 wt % salinity); [E] Yang et al.^125^ (300.15 K & 64,160 mg L^–1^ Reservoir (Weyburn) brine); [F] Wang et al.^174^ (311.15 and 0.25N NaCl); [G] Li et al.^180^ (313 K & 1 M NaCl + KI); and [H] Tang et al.^181^ (355.65 K & 243,143 mg L^–1^ Reservoir brine).

Figure 7 shows the effect of pressure on the diffusion coefficient of CO_2_ in various brines. A mixed picture is obtained regarding the effect of pressure. While the data of Tang et al.^181^ indicate that the CO_2_ diffusivity decreases as the pressure increases (at constant temperature and salinity), the opposite conclusion is reached when examining the data of Yang et al.,^125^ Shu et al.,^178,179^ Zhang et al.,^182^ and Wang et al.^174^ This discrepancy could be resolved by (i) either performing a new series of experiments or (ii) performing an extensive series of molecular dynamics simulations. From Figure 7, the majority of the experimental measurements for the CO_2_ diffusivity fall in the range 10^–9^–10^–8^ m^2^ s^–1^, there are also measurements in the range 10^–10^–10^–9^ m^2^ s^–1^ (e.g., Yang et al.^125^ using the dynamic interfacial tension method; Zhang et al.^182^ using the pressure decay method). A systematic study at the molecular level could shed additional light into this discussion.

Figures 6 and 7 clearly show that the diffusion coefficient of CO_2_ in brines depends on temperature, pressure, and salinity. Nonetheless, as a result of a lack of systematic experimental measurements (completely covering the three parameter space), a scarcely populated parameter space is currently available.

**Figure 8:**
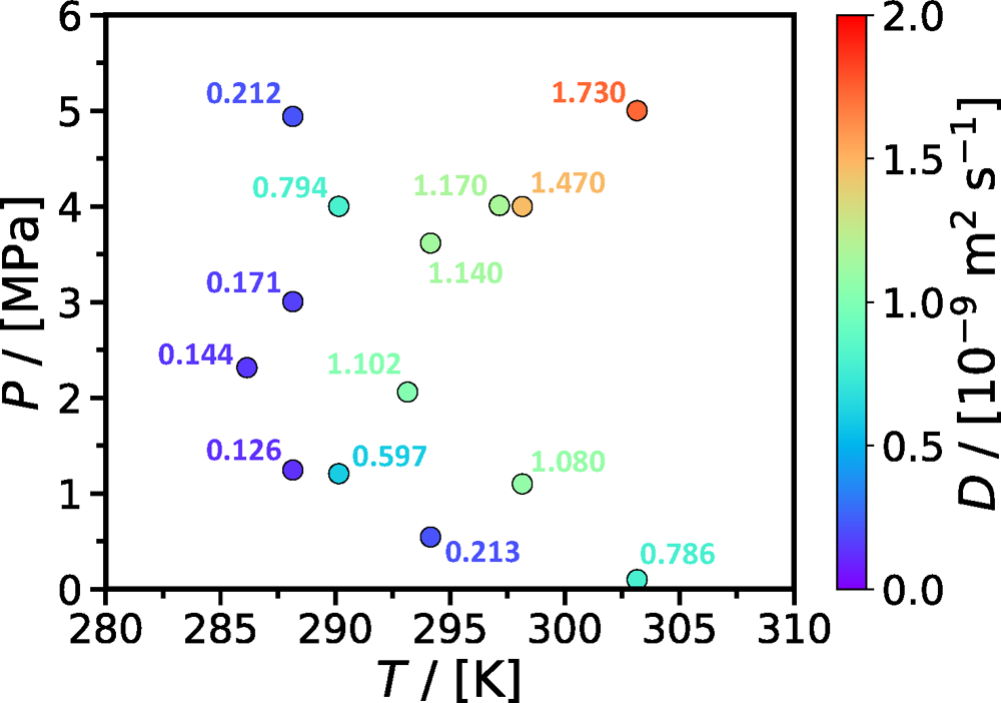
Combined effect of temperature and pressure on the diffusion coefficient of CO_2_ in oceanic brine (3 wt %). Symbols denote the experimental data of Zhang et al.^182^

Motivated by the emerging application of CO_2_ oceanic storage, Zhang et al.^182^ performed a systematic study of CO_2_ diffusion in brines (3 wt %) under various offshore conditions covering a temperature range of 286.15–303.15 K and a pressure range 0.1–5 MPa. The *P*, *T* conditions examined cover different oceanic depths. For a scenario of oceanic sequestration, the pressure and temperature profile will change as the oceanic depth changes (i.e., the pressure increases while the temperature decreases as the depth of the water column increases). Figure 8 shows the combined effect of pressure and temperature on the CO_2_ diffusion coefficient. The authors concluded that the influence of the pressure on the CO_2_ diffusivity was stronger at the higher temperatures considered.

#### 2.1.8 Convection-Enhanced Effective Diffusion Coefficients

While the molecular diffusion of CO_2_ in pure H_2_O or brines (in bulk or under confinement) is the primary focus of this review, natural convection-induced enhanced diffusion is briefly discussed in this section.

For constant temperature and salt concentration, as the pressure increases, the CO_2_ solubility in H_2_O increases as well.^41,44^ Yang and Gu^149^ reported that the density of CO_2_-saturated brine increased linearly with CO_2_ concentration. Consequently, as CO_2_ initially gets transferred through the gas–liquid interface, and subsequently dissolves into the brine, a density gradient evolves in the brine phase, as a result of the concentration gradient. Namely, the brine near the interface becomes heavier than the brine further away from the interface. Instability is created in the brine which results in a natural convection flow in the brine phase. This mechanism of forced mixing results in an accelerated mass transfer of CO_2_ in the brine under reservoir conditions (i.e., higher pressures). Often in the literature, this behavior has been interpreted using Fick’s second law:
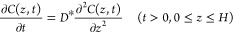
4where *C*(*z*, *t*) is the CO_2_ concentration in the fluid, *H* is the height of the fluid, and D* is a characteristic diffusion coefficient to be further discussed below. Equation 4 is subject to the following initial (eq 5) and boundary conditions (eqs 6 and 7). The initial condition is
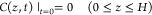
5

The boundary condition (B.C. 1) at the CO_2_-liquid interface is
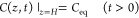
6where *C*_eq_ is the equilibrium CO_2_ concentration at the interface.

The boundary condition (B.C. 2) at the bottom of the cell is
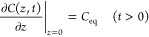
7

By combining the solution of the diffusion equations shown above with a mass balance, an expression can be developed which connects the pressure evolution of the diffusion process with time. If pressure decay experiments are available, then by plotting the curve:
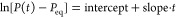
8we can obtain the diffusivity D* from the slope of eq 8 (slope = π^2^D*/(4*H*^2^)), where also intercept is a function of various parameters associated with the diffusing system.

**Figure 9:**
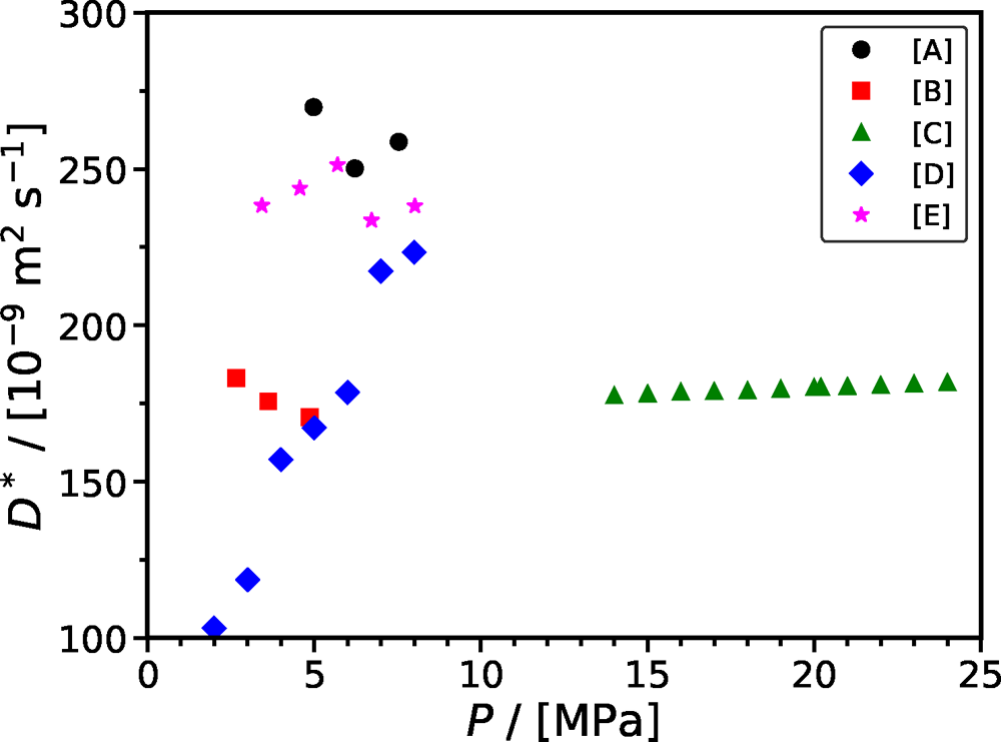
Effective diffusion coefficient, D*, of CO_2_ in different brines. Legend: [A] Yang and Gu^149^ (331.15 K & 4310 mg L^–1^ reservoir brine), [B] Yang and Gu^149^ (300.15 K & 4310 mg L^–1^ reservoir brine), [C] Tang et al.^181^ (355.65 K & 243 143 mg L^–1^ reservoir brine), [D] Li et al.^180^ (313 K & 1 M NaCl + KI), and [E] Wang et al.^176^ (318.15 K & 6,778 mg L^–1^ reservoir brine).

Figure 9 shows a number of different experimental studies that followed such an approach. The CO_2_ diffusivity values, D*, are more than ca. 2 orders of magnitude higher than the rest of the experimental data that have been discussed earlier in Figure 7. This is due to the fact that D* is an effective diffusivity which accounts for the combined effect of both molecular diffusion and natural convection on the mass transfer of CO_2_ in the liquid phase. To apply the methodology explained earlier, Wang et al.^176^ limited their analysis to the early time values from their pressure-decay experiments instead of the late-time. Even though their pressure decay measurements extended to more than 100 min, the analysis was limited to the first 40 min. At approximate that time, CO_2_ arrived at the closed end of the cell, and therefore the assumption that the liquid medium is infinite (i.e., an assumption required for the analytical solution of the diffusion problem) is not valid any more. Consequently, at the initial stages of the process, the calculated effective diffusivity includes both molecular diffusion and natural convection effects.

To accurately model the complex mass-transfer process (i.e., accounting for both the molecular diffusion and the natural convection), the diffusion equation with molecular diffusivity has to be solved simultaneously with the Navier–Stokes equation, which is essential for the description of fluid flow due to natural convection. Nevertheless, this approach requires the solution of a complex numerical problem.

### 2.2 Molecular Simulations

The versatility of MD simulations has been proven in literature for computing the self-diffusivity of CO_2_ in various solvents, such as aqueous alkanolamine solutions,^77,78,183^ ionic liquids,^184,185^ and deep eutectic solvents.^186,187^ MD simulation is a powerful method for the computation of diffusion coefficients of CO_2_ in H_2_O that can compliment experimental measurements and provide useful insight into the physical mechanisms governing diffusion at the nanoscale. MD often take less time and are less expensive than experiments, providing researchers with quicker means of studying diffusion phenomena.^45,188^ MD simulations eliminate safety concerns associated with high-pressure and high-temperature experimental setups.^45,78^ Furthermore, MD simulations provide the flexibility to ignore reactions between CO_2_ and H_2_O, enabling the focus on the diffusion without considering reaction products.^77,189,190^ Nevertheless, MD simulation results should always be validated against experimental data to ensure accuracy and reliability in predicting diffusion coefficients under different conditions. To validate computed diffusivities, comparisons with availalble experimental data are performed. In the absence of experimental diffusivities, researchers often resort to assessing agreement between the computed and readily accessible experimentally obtained thermodynamic and transport properties, such as densities and viscosities.^77,78,183^

#### 2.2.1 Simulation Methods

The computation of diffusivities can be achieved through either nonequilibrium MD (NEMD) or equilibrium MD (EMD) simulations.^64,65,191^ NEMD involves simulating the response of molecular systems to external perturbations. The results in NEMD simulations are heavily dependent on the specific applied external perturbation.^64,192^ Because of this reason, EMD simulations are commonly preferred for computing the diffusivity of CO_2_ in H_2_O.^45,73,191,193^ Two different methods can be used within EMD simulations to compute diffusivities: (i) The Green–Kubo method which involves integrating the velocity autocorrelation function over time, with this function slowly converging to zero.^64,65,194^ (ii) The Einstein relation which establishes a linear relationship between time and the mean-square displacement (MSD) of molecules to determine diffusivity.^64,65^ This linear relation is valid when the slope of mean-square displacement as a function of time equals 1 in a log(*t*)-log(MSD) plot. Open-source MD software for computing transport properties is available. The most widely used codes are GROMACS^195^ and LAMMPS.^83^ Recently, Jamali et al.^47^ developed the OCTP plugin for LAMMPS which allows the on-the-fly computation of diffusivities in MD simulations. Additionally, postprocessing tools such as PyLAT^196^ can be used to compute diffusivities using the molecular trajectories generated by MD simulations.

#### 2.2.2 Force Fields

In MD simulations, the so-called force fields play a crucial role since they provide the necessary description of the interactions between atoms and molecules within a system.^64,65^ Essentially, force fields describe the functional forms of the nonbonded potentials (e.g., van der Waals and electrostatic interactions) and bonded potential (i.e., bond stretching, angle bending, and dihedral rotations), allowing researchers to model the behavior of a molecular system. The accuracy and reliability of an MD simulation heavily depends on the accuracy of the chosen force field. Consequently, a well-parametrized force field is essential for obtaining meaningful insights into the structural and dynamic properties of molecular systems in silico.^64,65^

Although numerous force fields have been developed for CO_2_, the EPM2 force field by Harris and Jung^197^ and the TraPPE force field by Potoff and Siepmann^198^ are the most used for computing the diffusivity of CO_2_ in H_2_O. Both of these force fields include Lennard–Jones (LJ) interaction sites and point charges on the mass-centers of carbon and oxygen atoms of CO_2_. The point charges represent the quadrupole moment of CO_2_ (experimentally^199^ −4.3 × 10^–26^ esu) and the computed quadrupole moment of both of these force fields agree with the experimental value within the statistical uncertainty (for EPM2^197^ – 4.1 × 10^–26^ esu and for TraPPE – 4.52 × 10^–26^ esu).

In both EPM2 and TraPPE force fields, the C–O bonds are rigid. The C–O–C angle in the TraPPE force field is rigid, while in the EPM2 model, it is flexible (although the differences in the vapor liquid equilibria (VLE) and critical properties computed using a rigid angle and a flexible one are small).^197^ TraPPE uses the Lorentz–Berthelot combining rules (arithmetic mean for σ and geometric mean for ϵ), while in the EPM2 force field, a geometric mean is also used for the σ parameter of unlike atoms. The EPM2 force field was fitted to the VLE and critical properties of pure CO_2_. The change in the combining rule of the σ parameter between unlike atoms has a subtle impact on the σ parameter in interactions involving CO_2_ and H_2_O. For example, using arithmetic and geometric means, the computed σ parameters for carbon (EPM2 CO_2_)^197^ and oxygen (TIP4*P*/2005 H_2_O)^85^ are 2.957 95 Å and 2.951 11 Å, respectively. Although the EPM2 force field consistently underestimates the liquid phase densities by 1–2% between 221–289 K, the predicted VLE, critical temperature (within 3% of experimental value), critical density (within 4% of the experimental value), and critical pressure (within 1% of the experimental value) are in good agreement with experiments.^197,200^ The TraPPE force field was parametrized to reproduce the VLE of binary *n*-alkane/CO_2_ mixtures, specifically the propane/CO_2_ mixture.^198^ The VLE of pure CO_2_ is accurately captured by the TraPPE force field,^198^ demonstrating a good agreement between predicted and experimental densities. Notably, there is a slight overestimation in liquid densities and a minor underestimation in gas phase densities according to the force field predictions. The TraPPE force field exhibits excellent accuracy in predicting the critical properties of CO_2_, with agreement within 1% for critical temperature and density, and within 4% for critical pressure when compared to the experimental values.^200^ This shows the reliability of the TraPPE force field in capturing central thermodynamic properties.

Het Panhuis et al.^201^ developed a new force field which adopts the LJ parameters for carbon and oxygen atoms from GROMOS,^202^ while partial charges were fitted to reproduce the quadrupole moment of CO_2_, similar to the approach used for the point charges in the EPM2 force field,^197^ resulting in comparable point charges for these two force fields. Other force fields such as CHARMM27,^203^ COMPASS,^204^ and the force field from Merker et al.^205^ have also been used to model pure CO_2_ and mixtures.

An alternative method of designing a force field for accurately capturing the LJ interactions between CO_2_ and H_2_O involves using a specific set of LJ cross-interaction parameters rather than conventional mixing rules such as the Lorentz–Berthelot rules.^65^ Given the low solubility of CO_2_ in H_2_O, this approach proves particularly advantageous, ensuring that the calculated properties of pure CO_2_ and pure H_2_O remain unaffected. The study by Orozco et al.^206^ exemplifies this strategy by tailoring the LJ cross-interaction parameters between CO_2_ and H_2_O to achieve excellent agreement between the computed vapor–liquid equilibrium curve of CO_2_/H_2_O mixtures and experimental data. Vlcek et al.^207^ used a similar approach to optimize the cross-interaction parameters between CO_2_ and H_2_O to reproduce the mutual solubility of CO_2_ and H_2_O. Vlcek et al.^207^ showed that the optimized parameters were able to accurately reproduce the self-diffusivities of CO_2_ in H_2_O in a temperature range of 298–353 K and 0.1 MPa. In a different, yet related context, Costandy et al.^208^ used a modification factor (i.e., χ = 1.08 for TIP4P/Ice water model,^209^ and χ = 1.13 for TIP4*P*/2005 water model)^85^ to correct the Lorentz–Berthelot cross interaction energy parameter for the oxygen atom in the CO_2_ molecule and the oxygen atom in H_2_O. This approach has been used successfully for hydrate-related calculations in both MD^208,210^ and MC simulations.^211^

As one of the most important solvents in industrial and environmental processes, many different force fields have been developed for H_2_O. A few examples are SPC by Berendsen et al.,^212^ SPC/E by Berendsen et al.,^213^ TIP4*P*/2005 by Abascal and Vega,^85^ OPC by Izadi et al.,^214^ and TIP5P by Mahoney and Jorgensen.^215^ Polarizable force fields have also been developed for H_2_O^216−218^ and CO_2_.^87,219^ Such models can be more accurate in predicting phase equilibria and transport properties of pure components and mixtures, however, as they have not been used to compute the intradiffusivity of CO_2_ in H_2_O, further discussion is not provided in this review. A detailed discussion on polarizable and nonpolarizable H_2_O force fields falls outside the scope of this review. For more information about H_2_O force fields the reader is referred elsewhere.^85,212−215,220−223^ Nevertheless, it is crucial to exercise caution when selecting a force field for H_2_O to compute the self-diffusion coefficient of CO_2_ in H_2_O. Given the low solubility of CO_2_ under ambient conditions,^42^ the force field for H_2_O determines the density and viscosity of the solution. The self-diffusion coefficients of solutes and the solvent largely depend on these properties.

#### 2.2.3 Self-Diffusivity of CO_2_ in H_2_O at Ambient Pressure

**Figure 10:**
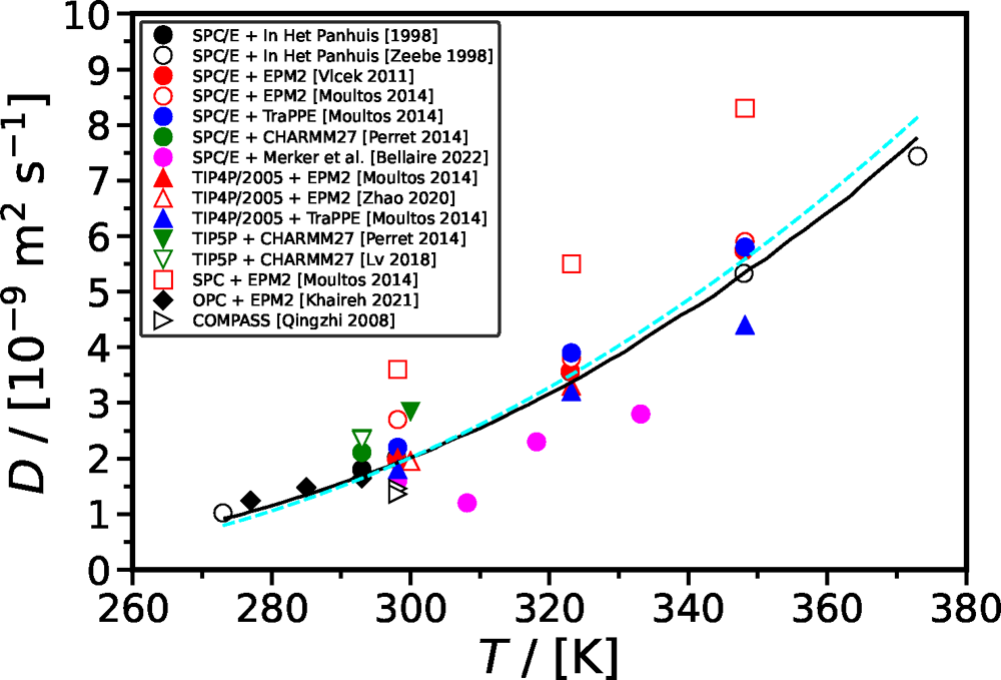
Intradiffusion coefficients of CO_2_ in H_2_O computed using different force fields^40,45,120,163,193,201,207,224−226^ as a function of temperature at 0.1 MPa. The black solid line and cyan dashed line represent the correlation developed by Mutoru et al.^61^ and the correlation developed in this work (SA-type), respectively.

**Figure 11:**
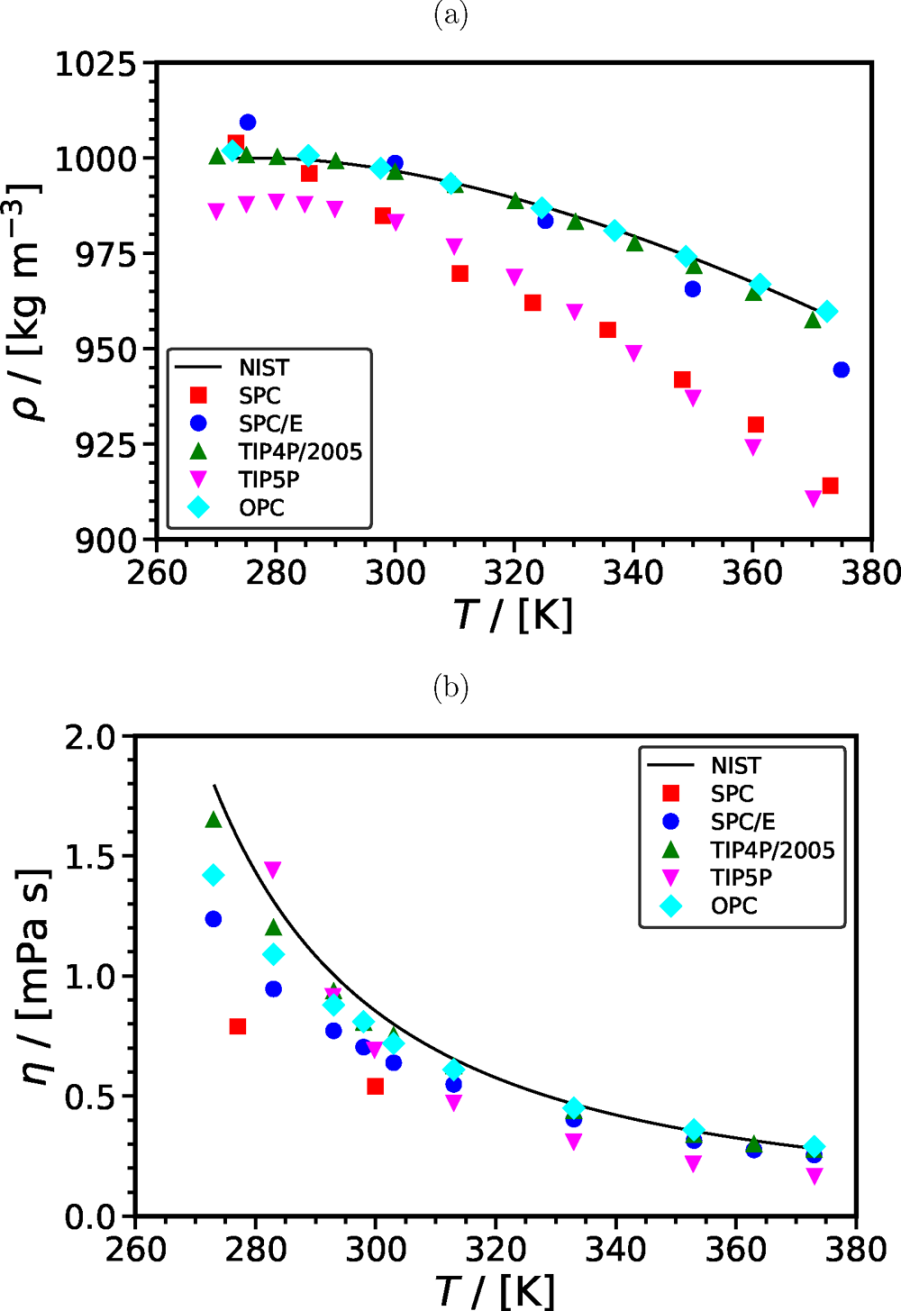
(a) Densities and (b) viscosities computed using SPC,^227,228^ SPC/E,^229−232^ TIP4*P*/2005,^230,233^ TIP5P,^233,234^ and OPC^214,235^ force fields for H_2_O and their comparison with the values from NIST database^236^ as a function of temperature at 0.1 MPa.

Figure 10 shows the self-diffusivity of CO_2_ in H_2_O computed using different force fields^40,45,120,163,193,201,207,224−226^ along with the correlations from Mutoru et al.^61^ and from this work (SA-type) as a function of temperature at 0.1 MPa. The self-diffusivities of CO_2_ computed in SPC H_2_O is the highest when compared with other force fields for H_2_O and do not agree with the experimental correlations. As shown in Figure 11, SPC force field significantly underestimates densities (up to 5% deviation from experiments) and viscosities (up to 32% deviation from experiments) of H_2_O. This implies that the SPC force field overestimates the free volume in the solution, leading to a significant overestimation of the self-diffusivities of the gas solute (i.e., CO_2_). The self-diffusivities of CO_2_ computed in TIP5P H_2_O are slightly higher than those computed in SPC/E H_2_O, and those in SPC/E H_2_O are higher than in TIP4P/2005 H_2_O. This pattern can be attributed to the fact that TIP5P underestimates the density and viscosity of the solution to the greatest extent, followed by the SPC/E force field and then TIP4P/2005 (Figure 11). The self-diffusion coefficients of CO_2_ computed with the EPM2 and TraPPE force fields are very similar, with TraPPE yielding slightly higher values than EPM2. The self-diffusivities of CO_2_ in H_2_O computed with the same force field are generally consistent throughout literature, except in the studies by Vlcek et al.^207^ and Moultos et al.^45^ In both these studies, SPC/E and EPM2 force fields were used for H_2_O and CO_2_, respectively. Vlcek et al.,^207^ however, computed the self-diffusivity of CO_2_ as 1.98 × 10^–9^ m^2^ s^–1^ at 298 K and 0.1 MPa, while at the same conditions, the self-diffusivity of CO_2_ was computed as 2.7 × 10^–9^ m^2^ s^–1^ by Moultos et al.^45^ The differences between the computed self-diffusivities by Vlcek et al.^207^ and Moultos et al.^45^ decrease with increasing temperature. This difference may be originating from the fact that Vlcek et al.^207^ used 512 molecules in total in their simulations and did not apply finite-size corrections. Finite-size effects were investigated by Moultos et al.^45^ and found to be negligible for the system size used, i.e., 2000 molecules. Later in this review, a detailed discussion on the finite-size effects on the diffusivities of CO_2_ in H_2_O is provided.

Overall, the data from literature suggest that the force field selection for H_2_O has a predominant influence on the self-diffusion of CO_2_ in H_2_O, particularly given the low concentration of CO_2_ in the solution due to its limited solubility in H_2_O. This is in line with MD studies of other gases diffusing into H_2_O, e.g., see Tsimpanogiannis et al.^72^ for the cases of H_2_ and O_2_. At ambient pressure, compared to the experimental correlations shown in Figure 10, the best performing combination of force fields are TIP4*P*/2005-EPM2 for *T* < 323 K and SPC/E-TraPPE for *T* > 323 K.

#### 2.2.4 Self-Diffusivity of CO_2_ in H_2_O at High Pressure

**Figure 12:**
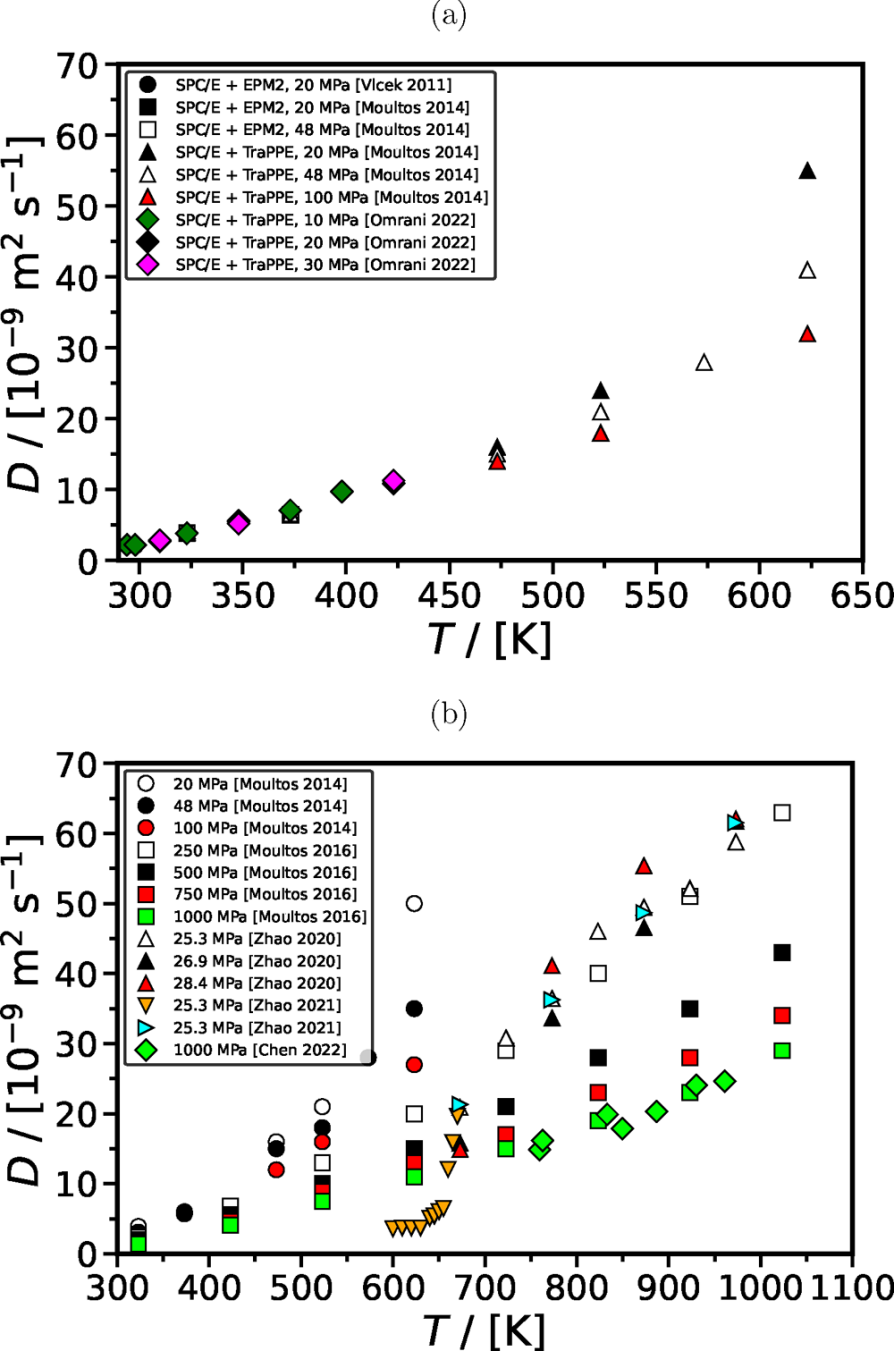
Self-diffusivities of CO_2_ computed using the (a) SPC/E force field for H_2_O and different force fields for CO_2_,^45,207,240^ and (b) TIP4*P*/2005 force field for H_2_O and EPM2 force field for CO_2_^45,73,193,241−243^ as a function of temperature and pressure.

At temperatures well below the critical point, the effect of pressure on the density and viscosity of the solution—and consequently on the self-diffusivity of CO_2_—is relatively minimal, given the low compressibility of the liquid phase. For example, at a temperature of 373.15 K, the viscosity of TIP4*P*/2005 H_2_O model demonstrates a slight 8% increase from 0.1 to 100 MPa.^237^ Similarly, the density of the TIP4*P*/2005 H_2_O model shows a 4% increase from 0.1 to 48 MPa at the same temperature.^45^ At elevated temperatures, however, the effect of pressure on the self-diffusivity of CO_2_ becomes more noticeable, as the solution exhibits higher compressibility under these conditions. For instance, at 1023.15 K, the density of TIP4*P*/2005 H_2_O increases by 69% from 250 to 1000 MPa.^73^Figure 12 shows the available self-diffusivities of CO_2_ from literature computed using MD simulations under high pressure. The data show that an increase in pressure causes a decrease in the self-diffusivities of CO_2_ in H_2_O. This becomes significant at *T* > 500 K. At 623.15 K, the self-diffusivity of CO_2_ in SPC/E H_2_O and TraPPE CO_2_ experiences a significant decrease (42%) over the pressure range from 20 to 100 MPa.^45^ This reduction in self-diffusivity closely aligns with a corresponding 40% increase in the density of the solution over the same pressure range at the given temperature.^45^

As shown in Figure 12, toward the critical temperature of H_2_O (experimentally 647 K^236^ while for TIP4*P*/2005 and SPC/E force fields, the critical temperatures are 623.3 K^238^ and 640 K,^239^ respectively), at ca. 22 MPa, the self-diffusivities of CO_2_ in H_2_O computed using all combinations of force fields for H_2_O and CO_2_ show a rapid increase. This is because the density of H_2_O changes rapidly toward the critical point.^239^ For higher pressures (*P* > 500 K), the computed self-diffusivities of CO_2_ in H_2_O show a linear increase with increasing temperature. Comparing the computed self-diffusivities of CO_2_ with experimental values is challenging given the scarcity of experimental data in the literature for high pressures and temperatures (relevant to CCS processes). As discussed earlier, the available experimental data by Lu et al.^116^ and Cadogan et al.^113^ is limited to temperatures up to 473 K and pressures up to 45 MPa. Moultos et al.^45^ computed the self-diffusivity of CO_2_ in H_2_O using the TIP4*P*/2005 force field for H_2_O and EPM2 for CO_2_ as 1.6 × 10^–8^ m^2^ s^–1^ at 473.15 K and 20 MPa. In comparison, Lu et al.^116^ experimentally measured the self-diffusivity of CO_2_ under the same conditions as 1.61 × 10^–8^ m^2^ s^–1^. The excellent agreement between the computed and experimental values, coupled with TIP4P/2005’s accurate predictions of H_2_O density at higher temperatures and pressures,^45^ suggests that the TIP4P/2005 and EPM2 force fields can be used for accurately predicting the intradiffusion coefficients of CO_2_ in H_2_O at elevated temperatures and pressures.

#### 2.2.5 Finite-Size Effects

MD simulations with periodic boundary conditions for the computation of self- and collective diffusion coefficients (as well as other properties such as activity coefficients^244^ and thermal conductivities)^245−247^ are susceptible to finite-size effects due to the long-range nature of hydrodynamic and electrostatic interactions.^248,249^ To obtain the diffusivities at the thermodynamic limit, it is necessary to extrapolate the computed diffusivities which scale with 1/*L* (1/*L* → 0, where *L* is the simulation box length).^250^ Commonly, the computed self-diffusivities are corrected with an analytical correction for finite-size effects derived by Yeh and Hummer:^251^
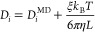
9where *D*_*i*_ is the self-diffusivity of species *i* at the thermodynamic limit, *D*_*i*_^MD^ is the self-diffusivity (or intradiffusivity) of species *i* computed from the MD simulation, ξ is a dimensionless constant equal to 2.837297 obtained by an Ewald-like summation of a periodic lattice, *k*_B_ is the Boltzmann constant, *T* is the absolute temperature, η is the viscosity computed from MD simulation, and *L* is the length of the simulation box. An extension of this correction was developed by Jamali et al.^54,252,253^ for mutual diffusivities. For an in-depth understanding of the finite-size effects, readers are encouraged to refer to the review paper by Celebi et al.^249^ and the work by Jamali et al.^54^

In MD literature reporting computations of CO_2_ in H_2_O intradiffusivities, the system sizes used in the simulations vary from a total of 216 molecules to 4124 molecules. Many of these studies do not correct the computed diffusivities for finite-size effects.^45,73,120,163,201,207,243,254^ In some of these studies, the diffusivities were computed using relatively big system sizes, and finite-size effects found to be negligible.^45,73,243,254^ However, in some studies, small system sizes were used, and thus, it is expected that the diffusivity computations are relatively innacurate. For example, In Het Panhuis et al.^201^ computed the self-diffusivity of CO_2_ in H_2_O at 293 K and 0.1 MPa as 1.8 × 10^–9^ m^2^ s^–1^ using 216 molecules in total and without correcting for finite-size effects. Considering eq 9 and the densities and viscosities shown in Figure 11, the finite-size corrected self-diffusivity of CO_2_ in H_2_O from this study is estimated to be 2.3 × 10^–9^ m^2^ s^–1^. This corrected value is 26% higher than the originally computed value by In Het Panhuis et al.^201^ Another example showcasing the importance of correcting for finite-size effects is found in the study by Vlcek et al.^207^ In their work, Vlcek et al. (using 512 molecules) computed the self-diffusivity of CO_2_ in H_2_O as 1.98 × 10^–9^ m^2^ s^–1^ at 298 K and 0.1 MPa. Interestingly, under identical conditions and using the same force field, Moultos et al. (using 2000 molecules) computed the self-diffusivity of CO_2_ as 2.7 × 10^–9^ m^2^ s^–1^.^45^ The inconsistency between these two studies can be attributed to the fact that Vlcek et al.^207^ did not account for finite-size effects, while using a relatively small system size. Considering eq 9, the finite-size corrected self-diffusivity of CO_2_ from Vlcek et al.^207^ is 2.33 × 10^–9^ (17.7% change due to finite-size effects) m^2^ s^–1^, aligning better with the value computed by Moultos et al.^45^ given the statistical uncertainty. From this discussion it becomes apparent that accounting for finite-size effects is crucial in MD simulations for accurately computing diffusivities, especially when rather small system sizes (<1000 molecules) are used.

#### 2.2.6 Transport Diffusivities of Aqueous CO_2_ Solutions

In most of the studies investigating the diffusivities of CO_2_ in H_2_O, the concentration of CO_2_ in the solvent is very low (1–5 molecules of CO_2_ in 216–4124 water molecules), as the solubility of CO_2_ in H_2_O under ambient conditions is quite low. At infinite dilution, the intradiffusivity of CO_2_ is practically equal to transport diffusion coefficients.^46,77,255^ A comprehensive study of transport diffusivities in aqueous solutions of CO_2_ was performed by Zhao et al.^242^ These authors^242^ computed the MS and Fick diffusivities (along with intradiffusivities) of aqueous solutions containing various gases, including CO_2_, for a temperature range of 673–973 K and a CO_2_ mole fraction range of 0.01–0.30. While the authors concluded that temperature and the concentration of CO_2_ in the solution significantly influence the MS and intradiffusivities, the interpretation of MS diffusivity trends with changing CO_2_ concentration remains challenging due to considerable scatter and uncertainties in the presented data (except for the data set at 673 K where a clear trend of increasing MS diffusivities with increasing CO_2_ concentration can be seen). Zhao et al.^242^ reported uncertainties up to 9% for MS diffusivities while the uncertainties for intradiffusivities were below 1%. For Fick diffusivities, Zhao et al.^242^ noted an increase with temperature and also suggested that the concentration of CO_2_ in the solution had no discernible effect on Fick diffusivities. The substantial scatter and uncertainties (up to 8%) in their Fick diffusivity data, potentially due to short simulation times (i.e., 1 ns), call for a careful interpretation.

Chen et al.^243^ also computed MS diffusivities of aqueous solutions of CO_2_ at 923 K and 25 MPa for a mole fraction range of CO_2_ between 0.005 and 0.900. Similar to the results from Zhao et al.,^242^ the MS diffusivities computed by Chen et al.^243^ show substantial scatter and large uncertainties (up to 16%, potentially due to low simulation times of 3 ns). The results from these authors, however, are shown to agree well with Darken and Vignes equations. Chen et al.^243^ argue that the composition effect of MS diffusivities comes with a trade-off between the number of hydrogen bonds per water and CO_2_ diffusivity, both hindering collective diffusivity in the solution. Increasing the mole fraction of CO_2_ in the solution decreases the average number of hydrogen bonds per water molecule, which increases the collective diffusivity. As the self-diffusivity of CO_2_ is lower than H_2_O, increasing the mole fraction of CO_2_ in the solution, decreases the collective diffusivity. In summary, while the studies by Zhao et al.^242^ and Chen et al.^243^ offered insights into MS and Fick diffusivities of aqueous CO_2_ solutions, further investigation including the solution structure with extended run times are essential for acquiring more robust and meaningful data.

#### 2.2.7 Correlations for the Diffusivity of CO_2_ in H_2_O from MD Simulations

**Figure 13:**
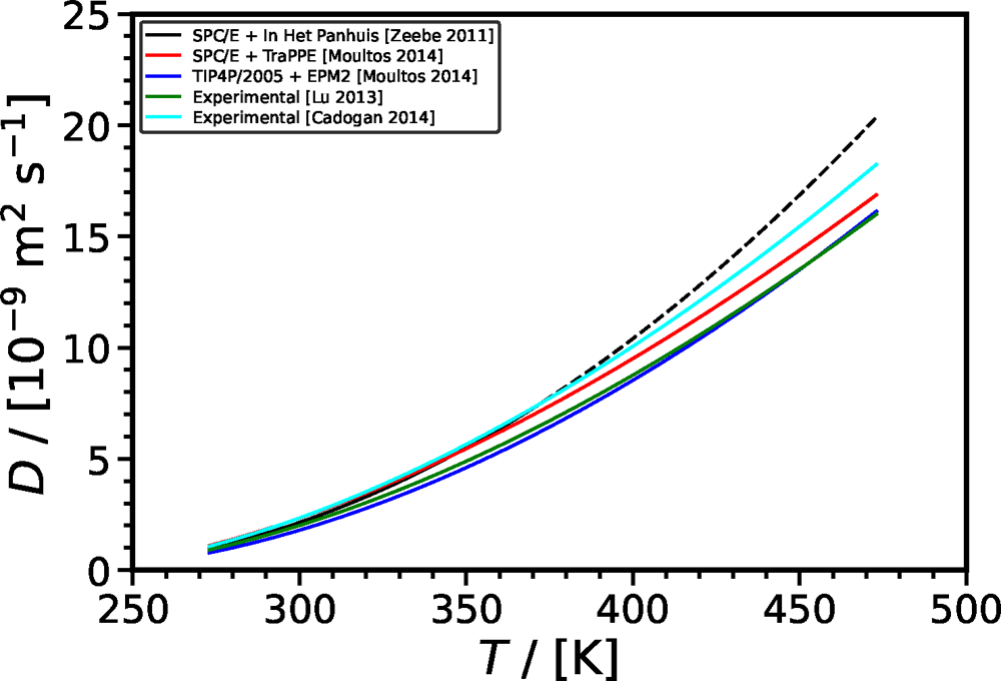
Correlations for the self-diffusivity of CO_2_ in H_2_O developed using the results from MD simulations^45,73,163^ and experimental data^113,116^ as a function temperature at 0.1 MPa. The solid lines represent the development temperature ranges of the correlations while the dashed lines show extrapolations to higher temperatures.

During the design and optimization of industrial processes (e.g., CCS, EGS, EOR), calculations rely on the assessment of thermodynamic properties, such as the diffusivity of CO_2_ in H_2_O, at different conditions. Despite the numerous advantages of MD simulations, their application to compute diffusivities across a very wide range of conditions is often impractical due to the long simulation times and supercomputers required. As a solution, various simpler correlations for the diffusivity of CO_2_ in H_2_O have been established in the literature.^45,73,163,193,241,242^ These correlations are derived from data obtained from MD simulations at different conditions, providing a more accessible and efficient means for estimating diffusivities at different conditions. As discussed earlier, the influence of pressure on the intradiffusivity of CO_2_ in H_2_O is negligible at low temperatures (*T* < 500 K). In literature, three pressure-independent correlations have been proposed for computing the self-diffusivities of CO_2_ in water at *T* < 500 K and 0.1 MPa.^45,163^ All these correlations have the functional form of eq 3. The comparison between the correlations developed using MD simulation data^45,163^ and the correlations developed using experimental results^113,116^ is shown in Figure 13. The first correlation, developed by Zeebe et al.^163^ for a temperature range of 273–373 K, used MD simulation results obtained with the SPC/E force field for water and the force field from In Het Panhuis et al.^201^ for CO_2_. Zeebe et al.^163^ demonstrated good agreement between their correlation, experimental values, and those calculated using the Stokes–Einstein equation. Moultos et al.^45^ developed two correlations based on MD data from the force field combinations of SPC/E + TraPPE and TIP4*P*/2005 + EPM2. While the results from these force field combinations were similar, Moultos et al.^45^ found that the correlation using data from SPC/E + TraPPE better aligns with the correlation from Zeebe et al.^163^ and the experimental correlation by Cadogan et al.^113^ The correlation derived from TIP4P/2005 + EPM2 agrees well with the experimental correlation by Lu et al.^116^ In Figure 13, the correlation from Zeebe et al.^163^ agrees well with the experimental correlation from Cadogan et al.^113^ within the temperature range of 273–373 K, however, when extrapolated to 473 K, it diverges from the experimental correlations. Both correlations by Moultos et al.^45^ exhibit strong agreement with experimental correlations in the temperature range of 273–473 K. The application of the correlations from Moultos et al.^45^ within this temperature range at 0.1 MPa is expected to result in more accurate self-diffusivities of CO_2_ in H_2_O.

**Figure 14:**
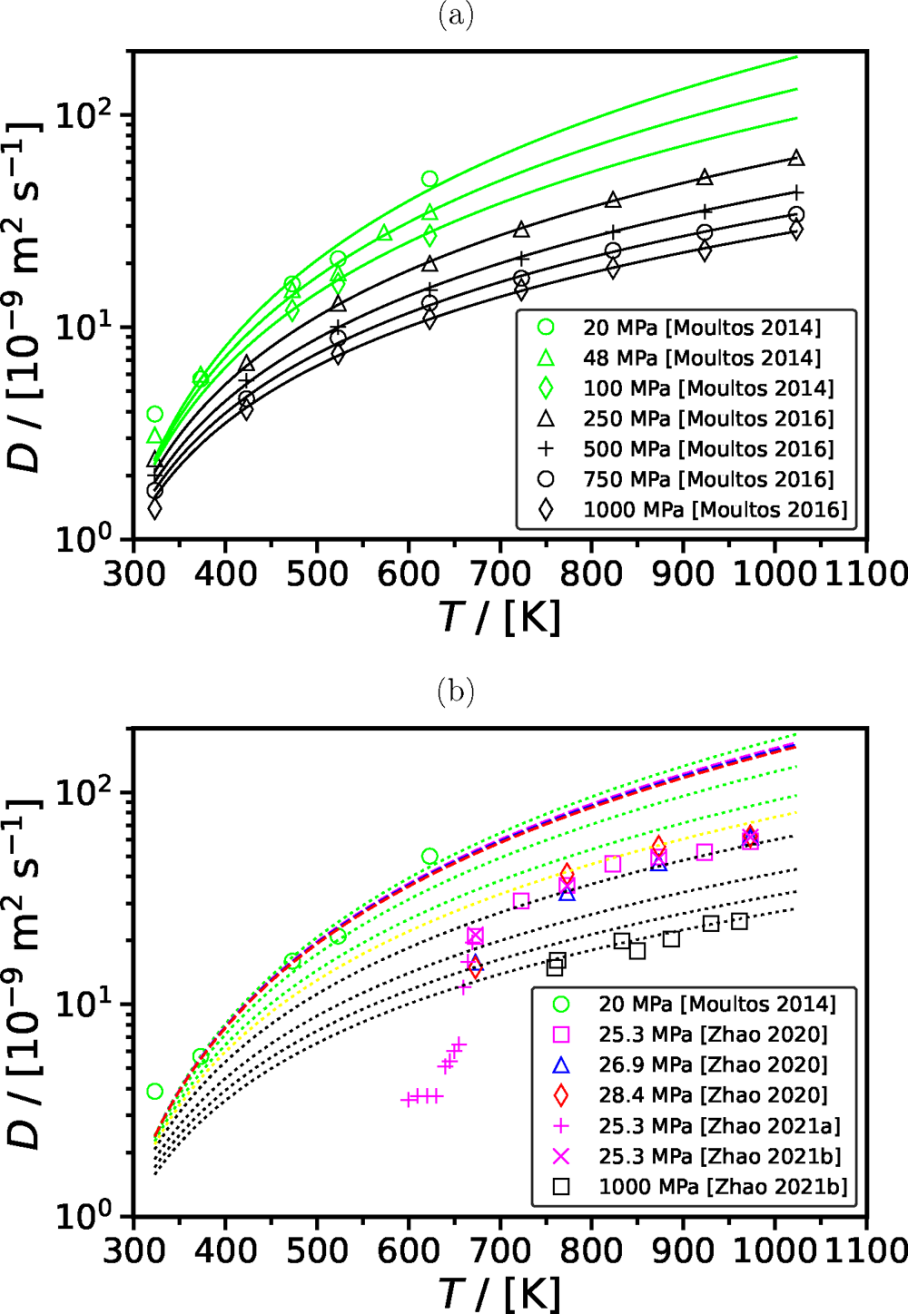
(a) Comparison of the MD data (denoted with symbols) and calculations (denoted with solid lines) using the pressure-dependent correlation of Moultos et al.^73^ Red symbols denote the data of Moultos et al.,^45^ while black symbols denote the data of Moultos et al.^73^ (b) Comparison of various MD data (denoted with symbols) and calculations (denoted with dotted/dashed lines) using the pressure-dependent correlation of Moultos et al.^73^ Notation for lines (from bottom to top): black dotted lines: 1000, 750, 500, and 250 MPa; yellow dotted line: 150 MPa; green dotted lines: 100, 48, and 20 MPa; and dashed lines: 28.4 MPa (red), 26.9 MPa (blue), 25.3 MPa (magenta).

In a subsequent study Moultos et al.^73^ used the TIP4P/2005 + EPM2 force field combination for the calculation of the diffusivity of CO_2_ in H_2_O at temperatures in the range 323.15–1,023.15 K and pressures equal to 250, 500, 750, and 1,000 MPa. The computed data were correlated with a Speedy-Angel type of equation (eq 3). In order to account for the effect of high pressures, the parameters *D*_o_ and *m* were given as functions of pressure (in MPa). Namely,
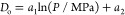
10and
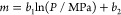
11where *a*_1_ = −2.3097 × 10^–9^, *a*_1_ = 2.1064 × 10^–8^, *b*_1_ = −0.17812, and *b*_1_ = 2.59406. This correlation, as clearly shown in Figure 14(a), describes the MD data very accurately at high pressures and temperatures. Furthermore, this correlation has been extrapolated to lower pressures, and compared against the MD data of an earlier study by Moultos et al.,^45^ at temperatures up to 623 K and pressures equal to 20, 48, and 100 MPa. Very good agreement was observed with these MD data, as well. Some deviations were observed for low temperatures, where the correlation reported by Moultos et al.^45^ should be used.

Two different correlations were developed for predicting the self-diffusivity of CO_2_ in H_2_O at higher temperatures (water at near- and supercritical conditions). Zhao et al.^193^ developed a temperature, density, and viscosity-dependent correlation developed at 673–973 K and 25.33 MPa for the self-diffusivities of H_2_, CH_4_, CO, O_2_, and CO_2_ in supercritical H_2_O expressed as
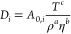
12where *A*_0,*i*_ is a gas specific constant (2.0078 × 10^–8^ for CO_2_), ρ is the density, η is the viscosity, and *a*, *b*, *c* are the respective exponents characterizing the effect of density, viscosity, and temperature on the self-diffusivity of these gases in supercritical H_2_O (*a* = 0.44, *b* = 1.42, and *c* = 2.76). The higher exponent on temperature suggests that temperature has the strongest effect on self-diffusivities, with the trend following *T* > η > ρ. To validate this correlation, Zhao et al.^193^ computed the self-diffusivities of CO_2_ under various conditions (673 to 973 K and 26.85 MPa, and 673 to 973 K and 28.37 MPa) using MD simulations and compared the results with those predicted by the correlation. Another correlation by the same group,^241^ applicable to near-critical H_2_O, was developed for the self-diffusivities of H_2_, CH_4_, CO, O_2_, and CO_2_ at a temperature range of 600–670 K and 25.33 MPa. While utilizing the same functional form (eq 12), different parameters were fitted to the data obtained using MD simulations (*A*_0,*i*_ = 4.7155 × 10^–3^ for CO_2_, *a* = 0.47, *b* = 1.2, and *c* = 1.01). The fitted values showed that viscosity has the most significant impact on intradiffusivities in near-critical H_2_O, distinguishing it from the supercritical conditions. Although the authors^241^ compared their results for H_2_ and O_2_ with other correlations from literature, they did not provide specific validation for the intradiffusivities of CO_2_, except for limited data from MD simulations at higher pressures. Validation for both of these correlations relies on data obtained through MD simulations. Direct comparison with experimental results is essential for assessing the reliability and predictive power of the correlations in capturing the real-world behavior of the self-diffusivity of CO_2_ in near-critical and supercritical water.

In 2021, Zhao et al.^242^ refined their correlation for the intradiffusivity of several gases, including CO_2_, in supercritical H_2_O. This enhanced correlation incorporated the effect of CO_2_ concentration in the solution, ranging from a mole fraction of 0.01 to 0.30. The functional form remains consistent with their prior studies (eq 12), but with additional factors accounting for solution composition and the thermodynamic factor. While these self-diffusivity correlations exhibit strong agreement with MD simulation data, direct comparisons with experimental data are challenging due to the limited availability of experimental results. Additionally, Zhao et al. extended their model to include MS and Fick diffusivities. For Fick diffusivities, the model relies solely on temperature and two gas-specific fitting parameters, while MS diffusivities are computed by dividing Fick diffusivities by the thermodynamic factor (which is a function of solution composition and temperature). As discussed earlier, the data presented by Zhao et al.^242^ for the MS and Fick diffusivities show substantial scatter and uncertainties, emphasizing the need for cautious interpretation regarding the model’s representation of reality.

Figure 14(b) shows the comparison between the calculations using the pressure-dependent correlation of Moultos et al.^73^ and the MD simulations reported by Zhao and Jin,^193^ Zhao et al.,^241,242^ and Chen et al.^243^ as a function of temperature and pressure. As can be clearly seen, the data of Chen et al. follow closely the correlation of Moultos et al. In sharp contrast, a large discrepancy is observed between the data of Zhao and Jin,^193^ and Zhao et al.^241,242^ Zhao and Jin^193^ and Zhao et al.^242^ reported data in the temperature range 673–973 K, while Zhao et al.^241^ reported data at 600–670 K (close to the H_2_O critical point). From Figure 14 it is evident that the data of Zhao and Jin^193^ and Zhao et al.^242^ fall in-between the correlation-lines corresponding to 150 and 250 MPa, while the simulations were performed in the range 25.3–28.4 MPa. Furthermore, for the MD data Zhao et al.,^242^ focusing on the proximity of the H_2_O critical point, the authors reported the diffusivity of CO_2_ in H_2_O at 620 K and 25.3312 MPa to be equal to 3.71 × 10^–9^ m^2^ s^–1^, while Moultos et al.^45^ reported a diffusivity value equal to 50 ± 4 × 10^–9^ m^2^ s^–1^ at 623 K and 20 MPa. Currently, the source of the discrepancy in not clear and further studies are required resolve it.

#### 2.2.8 Diffusivity of CO_2_ in Aqueous Electrolyte Solutions

**Figure 15:**
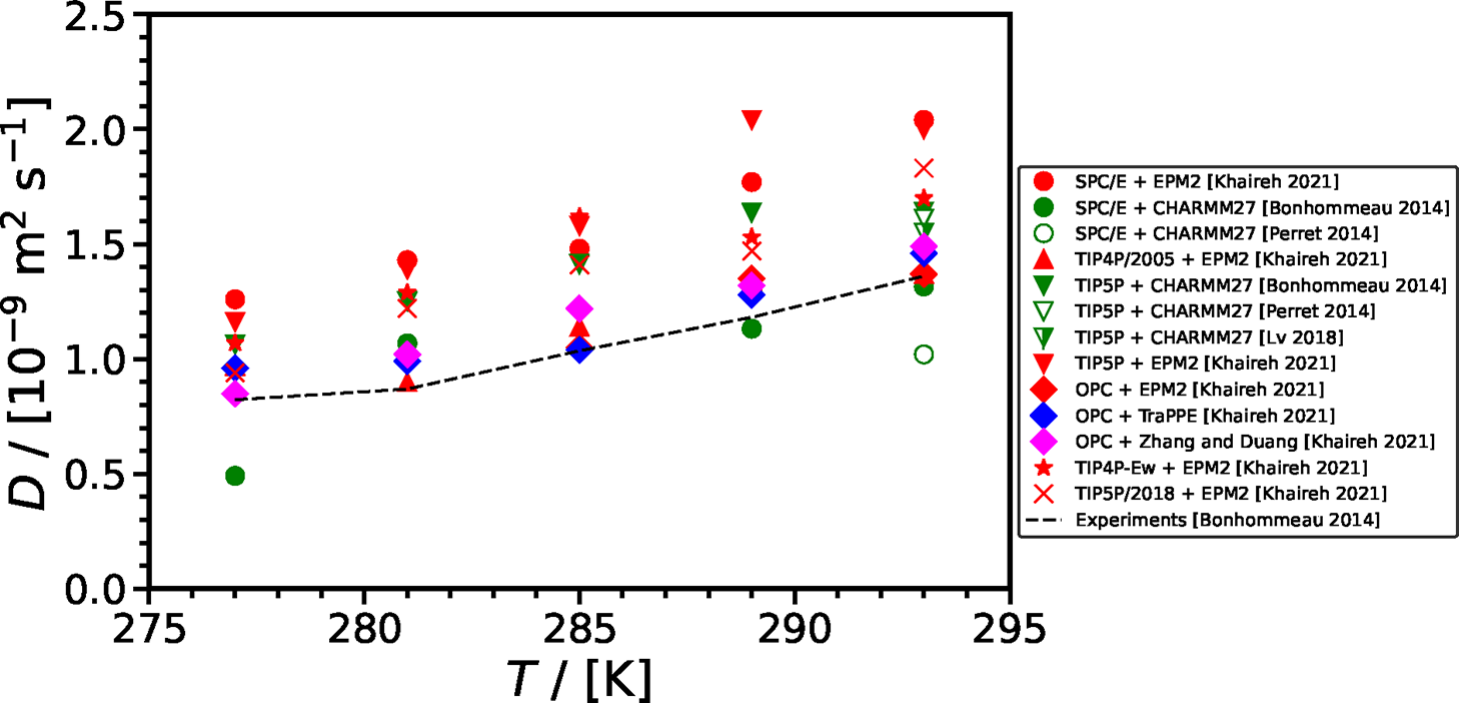
Comparison between computed^40,224,225,256^ and experimental^256^ self-diffusivities of CO_2_ in carbonated alcoholic drinks as a function of temperature at 0.1 MPa.

In Figure 15, intradiffusivity data from MD simulations^40,224,225,256^ are compared with experimental results from Bonhommeau et al.,^256^ in a carbonated hydroalcoholic solution (representing champagne) with mole fractions of CO_2_, ethanol, and H_2_O set at ca. 4.8 × 10^–3^, 0.042, and 0.95, respectively, at 0.1 MPa and a temperature range of 277–293 K. While MD studies using the same force fields generally exhibit consistent results, a discrepancy arises between the intradiffusivities of CO_2_ computed by Bonhommeau et al.^256^ and Perret et al.,^40^ specifically when SPC/E is used for water and CHARMM for CO_2_ and ethanol. Bonhommeau et al.^256^ argue that the improved equilibration method used in their study (replica exchange MD) is the cause of this discrepancy and their results are more accurate.

The investigation of Khaireh et al.^225^ shows the crucial role of the H_2_O force field in determining the intradiffusivities of CO_2_. The diffusivities computed using EPM2,^197^ TraPPE,^198^ and Zhang-Duan^257^ force fields are in agreement while the intradiffusivities computed using different water force fields show variations. The study by Lv et al.^224^ further emphasizes on this point, demonstrating agreement in the intradiffusivities computed in carbonated hydroalcoholic solution, cola (in this solution, ethanol was replaced with sucrose), and club soda (in this solution, ethanol was replaced with sodium bicarbonate) at 293 K and 0.1 MPa. Comparing with the experimental data from Bonhommeau et al.,^256^ OPC^214^ and TIP4P/2005^85^ H_2_O models exhibit excellent agreement throughout the temperature range of 277–293 K, while the other H_2_O force fields overestimate the self-diffusivity of CO_2_. This is expected since these force fields (OPC^214^ and TIP4P/2005^85^) represent H_2_O density and transport properties (viscosity and self-diffusivity of H_2_O) much better than the other H_2_O models (see Figure 11). We suggest the usage of OPC^214^ and TIP4P/2005^85^ force fields for the future MD studies, while caution is advised against SPC/E,^213^ TIP4P-Ew,^258^ TIP5P,^215^ and TIP5P/2018^259^ force fields.

Garcia-Ratés et al.^260^ investigated the diffusivity of CO_2_ in aqueous ionic solutions using MD simulations. The authors computed self- and MS diffusivities in brine for a temperature range of 333–453 K, a pressures range of 5–50 MPa, and a salinity range of 1–4 mol kg^–1^. The results^260^ showed that both self- and MS diffusivities increase with increasing temperature, while an increase in salinity from 1 mol kg^–1^ to 4 mol kg^–1^ led to a decrease of 34–41%. Typically to aqueous systems, the authors^260^ show that pressure has not a significant impact on the diffusivities. Additionally, Garcia-Ratés et al.^260^ developed a correlation linking MS diffusivities to self-diffusivities and rotational relaxation times, achieving a good agreement between predicted and computed MS diffusivities with an absolute average deviation of 15.4%. These findings contribute insight into the complex interplay of temperature, salinity, and pressure on the diffusivity of CO_2_ in brine which is relevant to CO_2_ sequestration in deep saline aquifers.

Understanding the diffusivity of CO_2_ in aqueous alkanolamine solutions is critical for absorption-based CO_2_ capture processes.^261^ Polat et al.^78^ investigated the temperature and alkanolamine concentration dependencies of infinitely diluted CO_2_ in aqueous monoethanolamine (MEA) solutions within a temperature range of 293–353 K and MEA concentrations ranging from 10 to 50 wt % using MD simulations. The results^78^ show a significant effect of temperature and MEA concentration on the self-diffusivities of CO_2_, with a 72–86% decrease in self-diffusivities from 10 wt % to 50 wt % MEA concentration in the solution. This study^78^ further revealed that the temperature dependence of the self-diffusivities in 10 wt % aqueous MEA solutions are higher than that in 50 wt % solutions. Similar observations were made by Yiannourakou et al.^183^ for CO_2_ in 30 wt % aqueous *N*-methyldiethanolamine (MDEA) solutions, demonstrating an increase in self-diffusivities from 2.50 × 10^–9^ m^2^ s^–1^ at 300 K to 1.03 × 10^–8^ m^2^ s^–1^ at 400 K. Polat et al.^77^ expanded the exploration to unloaded and loaded aqueous MDEA mixtures, showing that CO_2_ diffusion is 3.5 times faster in 10 wt % than in 50 wt % aqueous MDEA solutions within a temperature range of 288–333 K. Polat et al.^77^ attributed the slower diffusion of CO_2_ in concentrated MDEA solutions to stronger interactions between CO_2_ and surrounding molecules (both water and MDEA). Additionally, investigations^77^ into the self-diffusivities of CO_2_ in loaded 50 wt % aqueous MDEA solutions revealed a decrease with increasing CO_2_ loading, indicating that the CO_2_ capture with aqueous MDEA solutions slows down as CO_2_ loading increases. The research on CO_2_ diffusivity in aqueous alkanolamine solutions remains limited, focusing primarily on two alkanolamines and solely on self-diffusivities. The diffusivity of CO_2_ in aqueous solutions of other alkanolamines, such as diethanolamine (DEA) still remains unexplored, while comprehensive studies into collective diffusivities (Fick and MS) in CO_2_/H_2_O/alkanolamine mixtures are yet to be conducted, highlighting avenues for future research in the CO_2_ capture field.

## 3 Aqueous CO_2_ Diffusion in Confined Media

In applications such as gas separation and CCS in geological formations, CO_2_ molecules are constrained by confined media. The confinement effect imposes a heterogeneous distribution of the fluid in such a way that the thermophysical properties and structure are very different from an unconfined homogeneous fluid. For instance, the solubility of confined CO_2_ in H_2_O is different than that of the unconfined CO_2_ in H_2_O. When confined by hydrophobic surfaces, a higher solubility is expected due to the coadsorption of CO_2_ molecules, whereas a lower solubility is expected for the hydrophilic ones, because of the weak CO_2_–H_2_O interactions.^262,263^ Diffusion is also affected by confinement. Overall, CO_2_ diffusivity is expected to decrease because the mobility of molecules is reduced; preferential adsorption and steric hindrance may further decrease diffusion.^264^

### 3.1 Experimental Studies

#### 3.1.1 Experimental Measurement Techniques

Direct experimental measurements of the molecular diffusion of confined fluids are often infeasible or nontrivial.^265^ Nevertheless, trends can be observed through experiments, and macroscopic diffusion-related properties can be determined. Quasi-Elastic Neutron Scattering (QENS) can be used in combination with MD simulations to investigate the stochastic motion of molecules. From the scattering signal, one can devise a model based on functions, such as Lorentzian and Gaussian, and fit parameters to determine diffusion coefficients, residence times, and correlation lengths.^266^

The transport diffusivity of pure CO_2_ in silicalite has been studied with QENS and MD by Papadopoulos et al.^267^ The same order of magnitude was obtained by both methods, however, QENS diffusivities were higher at every condition studied. The trend with loading inside the zeolite was similar for QENS and MD. The dynamics of pure CO_2_ with QENS has been investigated in other confining materials such as the zeolite AlPO_4_-5^268^ and the metal–organic frameworks (MOF) MIL-140A(Zr)^269^ and UiO-66(Zr).^270^ The mixtures of CO_2_ with CH_4_,^268,271,272^ C_2_H_6_,^273,274^ and H_2_^269^ have also been studied. To the best of our knowledge, the only work available on the diffusion of the mixture CO_2_–H_2_O studied with QENS is from Hunvik et al.^275^ These authors investigated the dynamics of the hydrated interlayer of hectorite with and without CO_2_ using QENS techniques. The system has been dominated by jump-diffusion mechanisms, in which the molecule motion occurs via almost instantaneous jumps. Because individual molecule trajectories are indistinguishable, the system is characterized by a single random jump diffusion coefficient, a residence time, and a mean jump distance. Based on the diffusion parameters, the authors concluded that the dynamics in the interlayer of a hydrated smectite remains unchanged after exposure to CO_2_.^275^

Nuclear magnetic resonance (NMR) may be applied to investigate dynamic properties. By signal attenuation, one can fit a model to determine diffusion coefficients. Bowers et al.^276^ have shown with NMR that CO_2_ has a parallel preferential orientation when confined in the interlayer space of hectorites. The main CO_2_ dynamics are characterized by fast-motion rotation to the normal surface at rates ca. 10^5^ Hz. Peksa et al.^277^ investigated the diffusion of pure CO_2_ confined by DMOF-1 with the ^13^C pulsed field gradient (PFG) NMR technique. They discovered that CO_2_ is highly mobile in this MOF with diffusion trace tensor of (6.2 ± 1.0) × 10^–9^ m^2^ s^–1^. The anisotropy (ratio between the parallel and perpendicular diffusion coefficient) is equal to 3.^277^ Using similar techniques, Forse et al.^278^ have shown that this anisotropy is equal to ca. 30 for the CO_2_ diffusion in the Zn_2_(dobpdc) MOF. The diffusion coefficient of CO_2_ confined by pores of silica is at least 1 order of magnitude lower than in the bulk. By modifying the silica surface, further decrement in the CO_2_ diffusivity occurs due to higher adsorption.^279^ Despite the numerous NMR studies of CO_2_ diffusion in various confining materials,^276−281^ to the best of our knowledge no studies investigating the CO_2_–H_2_O mixture exist in the open literature.

Microfluidics can be applied along with fluorescence techniques to investigate CO_2_ diffusion in aqueous mixtures. By the spatial evolution of the pH measured by fluorescence emissions, one determines the CO_2_ concentration profile with time. The diffusion coefficient is obtained by fitting the profiles with analytical diffusion models.^121,282^ Sell et al.^121^ developed a microfluidic device capable of measuring diffusivity in less than 90 s. The authors determined the CO_2_ diffusion coefficient in a wide range of pressure (0.5 to 5 MPa) and salinity (0 to 5 M NaCl) and showed that their results are in good agreement with previous experiments and models: CO_2_ diffusivity is almost independent of the pressure, and decay exponentially with salinity. Peñas López et al.^282^ have investigated the CO_2_ radial diffusion from a CO_2_ bubble to an air-saturated H_2_O solution confined by a horizontal Hele-Shaw cell via pH-sensitive planar laser-induced fluorescence (PLIF). Different analytical models were able to successfully describe the diffusion-driven transport, and the characteristic length of the isoconcentration front evolves proportionally to , with *t* being time.

Finally, chromatographic techniques may also provide insights into macroscopic diffusion. Suzuki et al.^283^ performed chromatographic experiments in a zeolite bed with different humidity contents. The authors showed that the contribution of the macropore diffusion on the interparticle diffusion is dominant compared to the micropore diffusion. The CO_2_ interparticle diffusion in hydrophobic zeolites has shown no dependency on the moisture level.^283^

#### 3.1.2 Core Flooding Experiments

Core flooding experiments are a common approach when the effect of confinement, via a porous medium, on different thermodynamic or transport properties is of interest. For the case of CO_2_ diffusion in liquid H_2_O under confinement, core flooding experiments usually provide an effective diffusivity, which is different than the molecular diffusivity in bulk fluids, that also includes the effect of the porous medium.

Macroscopically, core flooding experiments may also provide insights related to CO_2_ transport in a confined environment, for instance, through the rock permeability calculation.^284−286^ Moortgat et al.^287^ have shown that, to represent the core-flooding experiments, the applied numerical model needs to take into account the CO_2_ Fickian diffusion. Busch et al.^288^ measured the effective diffusion coefficient of CO_2_ in a H_2_O-saturated shale sample under subsurface conditions by fitting the cumulative amount of CO_2_ passing through the pores to a nonstationary diffusion model. The diffusivity is estimated at 3.08 and 4.81 × 10^–11^ m^2^ s^–1^ for the first and second run of the experiment, respectively. The difference between runs is attributed to CO_2_ partial sorption in the first run. By comparing the effective diffusivity with the diffusion of CO_2_ in bulk H_2_O, the sample tortuosity is estimated to be between 40 and 70.^288^ Si et al.^289^ conducted a similar experimental study for the measurement of the effective diffusion coefficient of CO_2_ in water-saturated coal.

Renner^290^ used Berea cores and examined the diffusivity of CO_2_ in 0.25 N NaCl (i.e., 14.625 g per L H_2_O) brines at 311 K and pressures up to 5.86 MPa. The author concluded that for the chosen conditions there was no difference identified between diffusion coefficients measured for vertical or horizontal positioning of the cores (i.e., the gravity-induced convection had minimal effects on the measured diffusivities). Shi et al.^154^ reported experimental measurements for water-saturated or brine–saturated packs of two different porous materials. Namely, (i) 1.6 mm soda lime glass beads with 40% porosity and 250.11 × 10^–11^ m^2^ permeability, and 125–150 μm quartz particles with 45% porosity and 0.48 × 10^–11^ m^2^ permeability. Seyyedi et al.^291^ performed experiments in brines (0–20 wt %) at temperatures in the range 311.15–331.15 K, in a bead pack cell, with 37% porosity and 2.95× 10^–9^ m^2^ permeability. The authors used a mathematical model to account for the density-driven convection and investigated the effect of temperature and brine salinity on the convection mechanism. They reported that an increase in salinity results in reduction of the diffusion coefficient, while an increase in the temperature results in an increase of the diffusion coefficient, which is consistent with previous studies. Additionally, they reported that an increase in temperature or brine salinity has an unfavorable effect on the convection mechanism. Zhang et al.^182^ used Berea cores and examined the effect of the core permeability (10, 50, and 100 mD) on the CO_2_ diffusivity in brine-saturated (3 wt %) cores at *T*, *P* conditions equal to 290.15 K and 4 MPa, respectively. They reported effective diffusion coefficients of CO_2_ in the brine-saturated cores equal to 1.22 × 10^–15^, 3.87 × 10^–15^, and 4.81 × 10^–15^, m^2^/s for the aforementioned permeabilities, respectively. Li et al.^292^ reported effective diffusion coefficients in brine-saturated (0.5–2 mol L^–1^ NaCl) Berea cores. The authors examined temperatures in the range 313.15–373.15 K and pressures in the range 8.28–30.94 MPa, and provided empirical pressure–temperature-based correlations for the CO_2_ diffusivities in brines under reservoir conditions. Li et al.^150^ reported experiments using Berea and Benthiemer core samples. The authors introduced a new method for the measurement of effective gas diffusion coefficients in brine-saturated consolidated cores based on a radial diffusion model. To this purpose, mathematical models were developed to obtain the gas effective diffusion coefficient from the measured pressure decay curve. Li et al.^150^ concluded that the diffusive tortuosity factor of the examined cores was about 10. Basilio et al.^124^ used the pressure decay method to measure the molecular CO_2_ diffusion coefficients in pure water, at 293.15 K, using capillary tubes, packed with glass beads with three different grain size ranges: (i) 45–90 μm, (ii) 200–300 μm, and (iii) 425–560 μm. The use of capillary tubes in this experimental approach allows for the disregard of density-induced convection during the diffusion process. Moghaddam et al.^293^ used different unconsolidated sand packs with permeabilities in a range of ca. 3.1–2.546 m^2^ to measure the effective CO_2_ diffusion coefficients in pure water at 310.15 K. The experimental diffusivities were subsequently correlated with the dimensionless Rayleigh number.

#### 3.1.3 The Challenge of Comparing Experimental and Computed Diffusivities in Confined Media

The comparison of diffusion data from experiments and theoretical models is not always straightforward. The multiple definitions of diffusivities (e.g., self-, Fickian, Maxwell-Stefan) makes the comparison even harder, since one needs to be very careful on how the diffusion coefficient is defined, which depends on the proposed driving force (concentration, mole fraction, or chemical potential). Only few techniques, such as NMR^294^ and QENS^295^ can provide essential insight into diffusion mechanisms under confinement. More difficulties emerge when comparing real materials (with defects, different geometries, shapes, and crystallographic planes) with the simulations, which are usually carried out with perfect materials (e.g., no defects). Due to this, the experimentally determined diffusion tensor is different from the diffusion tensor computed with MD simulations. The latter is a diagonal tensor even for anisotropic materials, whereas the former exhibits off-diagonal components in anisotropic materials.^296^ This is a direct consequence of the spatial scale at which the experiment and the simulations are conducted. When confined in a idealized shape (e.g., slit, cylindrical, or spherical pores), the diffusion tensor of the fluid is necessarily diagonal.^296,297^ Another issue is related to Darcy’s law, widely applied to describe porous media flow. For highly confined media with low permeability (e.g., some nanoporous materials), due to the strong adsorption, Darcy’s law may fail, as shown via molecular simulations for kerogen.^298^ Such discrepancies between experiments and simulations could be tackled to some degree by a more systematic effort by the scientific community in determining diffusion coefficients, and transport properties more generally, in confined media.

### 3.2 Molecular Simulations

For a full description of the microscopic diffusion under confinement, molecular simulation techniques can be a very helpful approach. From MD simulations, we obtain the trajectories of the molecules from which the diffusion of each species can be computed. The initial configuration of MD simulations of confined fluids may be obtained with Grand-Canonical Monte Carlo (GCMC) simulations, in which the temperature *T*, the volume *V*, and the chemical potential of each species μ_*i*_ are fixed.^264,299−302^ At equilibrium, the chemical potential of confined species is equal to their chemical potential in the bulk, and the number of molecules is defined based on insertion/deletion techniques. Performing GCMC to generate initial configurations for MD simulations, one guarantees a confined fluid distribution that is in equilibrium with an unconfined fluid at the specified bulk pressure.

Via MD simulations, the diffusion of confined CO_2_ has been investigated as part of various mixtures, such as shale gas,^303^ CH_4_,^304−306^*n*-C_4_H_10_,^307^*n*-C_7_H_16_,^308^*n*-C_8_H_18_,^309^ and ionic liquids.^310,311^ The diffusivity of pure CO_2_ has also been investigated under confinement by different materials, such as MOF,^312−314^ graphene sheets,^315^ zeolites,^316^ calcites,^317,318^ silicalites^314,319^ and clays.^320^ In this review, we focus only on the results related to diffusion of the confined mixture of CO_2_ and H_2_O.

#### 3.2.1 Force Fields

**Figure 16:**
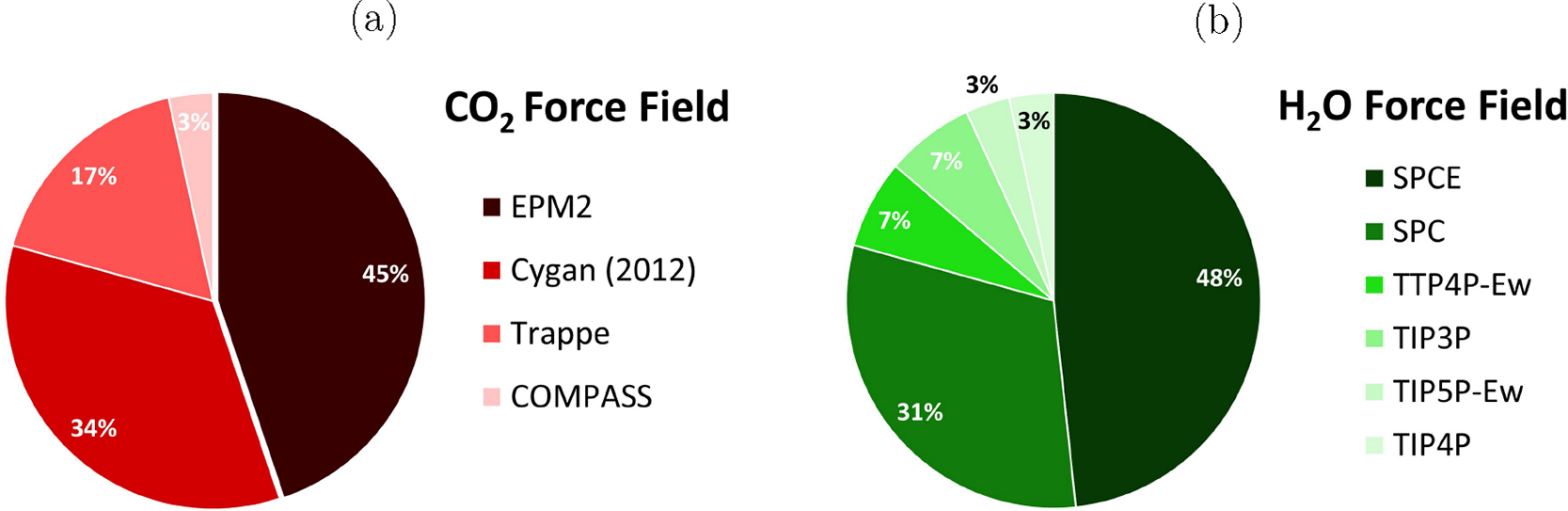
Overview of the relative popularity of (a) CO_2_ and (b) H_2_O force fields used in the literature for the computation of CO_2_–H_2_O diffusion in confinement.

As we extensively discussed earlier, in MD (and GCMC) simulations, accurate force fields are required for the description of the interactions between the species. Similarly to the bulk phase, to study transport properties of confined CO_2_–H_2_O mixtures, the EPM2^197^ and SPCE^213^ force fields for CO_2_ and H_2_O, respectively, are the ones more commonly used in the literature (see Figure 16). These force fields were developed to reproduce bulk properties at homogeneous conditions, and thus, they may not always be a good representation of the interactions of the molecules in confinement, especially taking into account the solid–fluid interactions. Cygan et al.^321^ have developed a fully flexible force field for CO_2_ based on vibrational data of confined CO_2_. This force field has been widely used to study CO_2_ diffusion in confinement.^263,264,301,322−328^

The choice of force field representing the confining material is also crucial. CLAYFF^329^ is the most used force field to represent natural confining media. CLAYFF has been shown to be suitable for representing hydrated minerals, such as hydroxides, oxyhydroxides, and clays, in contact with fluids. CLAYFF is based on metal–oxygen ionic interactions and the only bonded interactions are in the terminal groups.^329^ To investigate CO_2_ diffusion under confinement, CLAYFF has been used to represent the mineral structure of montmorillonite,^264,265,301,323,325,327^ hectorite,^330^ beidellite,^302^ forsterite,^331^ kaolinite,^262^ sepiolite,^328^ palygorkite,^328^ and hydrocalcite.^322^ To represent kerogen^332^ and calcite^263,333,334^ structures, COMPASS^204^ and the force field developed by Xiao et al.^335^ are the ones commonly used.

To represent artificial materials, various force fields can be used. For carbon-derived materials such as carbon nanotubes^300,336^ the LJ carbon is commonly represented by the chargeless FF from Steele.^337^ The CVFF^338^ has also been applied to model graphene sheets.^339^ This force field, however, has been originally parametrized to represent proteins.^338^ Sizova et al.^340^ applied the Steele FF^337^ combined with the OPLS-AA^341^ to represent, respectively, the carbon atoms and the functional groups in the structure of the CMK-5 mesoporous.

The representation of MOF usually is made by the generic DREIDING force field^342^ and the universal force field (UFF).^343^ Both these models have already been used to investigate CO_2_ diffusion.^344−347^ Bendt et al.^344^ devised a force field based on Density Functional Theory (DFT) calculations capable of better predicting the potential energy surface around the open metal sites of Mg-MOF-74.^348^ The authors have investigated the effect of accounting for flexibility in the solid framework on the CO_2_ diffusion. Although the adsorption energy in the flexible material is about the same as in the rigid one, the equilibrium distance between guest molecules and the open metal site is enlarged in the former, which increases the diffusivity of CO_2_ molecules when flexibility is taken into account.^344^

#### 3.2.2 Methods

When confined, the fluid density is no longer spatially homogeneous, and the diffusion coefficient exhibits a tensorial nature. Following Einstein’s method, diffusion coefficients may be obtained from the mean squared displacement evolution with time if the medium is homogeneous. For inhomogeneous fluids, however, Einstein’s equation is no longer valid, not only because of the inherent inhomogeneity, but also because Einstein’s solution to the mass balance equation is found by considering boundary conditions at infinity, which does not hold for confined systems. In this case, both parallel and perpendicular components of diffusion coefficients should be computed using other methods. For parallel self-diffusion coefficients, the method proposed by Liu et al.,^349^ based on the solution of the Smoluchowski equation and the calculation of the survival probability, is adequate and has been applied in the literature.^317,350^ Similar to Einstein’s relation, the method is based on the computation of diffusivity from the mean squared displacement, but, to account for the medium inhomogeneity, the mean squared displacement must be divided by the survival probability of molecules to stay in the reference layer in which the diffusivity is evaluated.

For the perpendicular self-diffusion coefficient, some methods have been proposed in the literature. Liu et al.^349^ proposed a method that requires two simulations in paralell, one of them using Langevin dynamics.^65^ Mittal et al.^351^ proposed a method based on a discretized version of Smoluchowski equation. The Mean First-Passage Time has been applied by von Hansen et al.^352^ to compute the diffusion of H_2_O in a lipid bilayer. Carmer et al.^353^ proposed the steady-state color reaction-counterdiffusion method. Finally, Franco et al.^297^ analytically solved the Smoluchowski equation deriving a method to compute the perpendicular self-diffusion coefficient. It is important to note that although these methods have been applied in the literature, many authors continue to apply Einstein’s relation to compute diffusion coefficients in confined media. Overall, the perpendicular component of CO_2_ diffusion is lower than the parallel one due to the constraints imposed by the surface in that direction.^262,326,327,339^ Usually, the diffusion coefficient tensor is dependent on the distance from the surface, in such a way that the Smoluchowski equation needs to be solved for each direction in layers parallel to the confining media. When a molecule goes from one layer to another, it no longer contributes to the calculation of the diffusion in its initial layer. This effect is accounted for by the survival probability of molecules in space.^349^

Using the method proposed by Liu et al.^349^ for the parallel self-diffusion coefficient, Chialvo et al.^354^ computed the H_2_O and CO_2_ self-diffusion coefficients parallel (*D*_∥_^s^) to a silica surface in a H_2_O-rich environment. They have computed *D*_∥_^s^ in both external and internal (confined) interfacial regions. Externally, the diffusion coefficient of H_2_O decreases monotonically with decreasing distance from the silica surface.^263,354^ In the confined region of hydrophobic surfaces, the diffusivity is no longer monotonic due to the local fluctuations of density and composition. Under severe confinement of hydrophobic silica (distance of 0.6 nm between plates), CO_2_ concentrates in a single peak in the middle of the pore and achieves a diffusivity (2.8 × 10^–9^ m^2^ s^–1^) close to the bulk value (3.2 × 10^–9^ m^2^ s^–1^). Santos et al.^334^ have also computed the parallel diffusion of CO_2_ with low H_2_O concentration at calcite and silica surfaces, accounting for the inhomogeneity of the confined fluid. All other studies available on the confined CO_2_–H_2_O diffusion have computed the diffusion coefficients from the slope of the mean squared displacement with time, following Einstein’s relation, which could lead to misleading conclusions and inaccurate results.

The self-diffusion coefficient relates to the thermal energy of particles through Brownian motion. The presence of other particles, especially a different component, may interfere with the particle motion. Transport diffusivity, such as Maxwell–Stephan or Fick diffusion coefficients, accounts for the influence of collective interactions on the fluid motion. Transport diffusion coefficients can be computed from EMD or NEMD. The former may converge very slowly because it needs to account for cross-correlation between all particles.^355^ Various nonequilibrium techniques may be applied to investigate diffusion flux under confinement. With gradient relaxation molecular dynamics (GRMD), an initial concentration gradient is established and the transport diffusivity is obtained by fitting the diffusion equation with the system relaxation with time.^356^ In the dual control volume grand canonical molecular dynamics (DCV-GCMD), two bulk reservoirs with distinct chemical potential are coupled to opposite edges of the confined system; the chemical potential gradient is kept constant via particle creation/destruction in the reservoirs, in such a way that a steady state flux is established and the diffusion coefficients can be obtained.^357^ An external field (EF-NEMD) can also be applied in the fluid particles to induce a mass flux in a predefined direction.^358^ Care should be taken because the effect of an external field on the interaction between particles may not be negligible.^355^

Magnin et al.^346^ have computed both self-and MS diffusivity of CO_2_ and H_2_O confined by a MOF using Einstein’s method (EMD) and applying a constant force on the guest molecules (NEMD), respectively. They found that , which indicates that for CO_2_ the cross-interaction effects on diffusion may be negligible compared to the strong effect imposed by the confinement. The same does not apply for H_2_O at all conditions: by increasing the pressure, and consequently the loading, the self-diffusivity deviates from the Maxwell-Stephan one, and collective interactions may no longer be neglected.^346^ Yang et al.^300^ related the self- and transport diffusion coefficients in the CO_2_–H_2_O mixture confined in carbon nanotubes (CNT). The authors have used the pure component sorption and diffusion data, and the saturation loading, and derived a loading-independent self-exchange coefficient. They found that the MS diffusivity of CO_2_ is almost independent of the loading. Overall, good agreement is obtained with the correlation. At low loading (or high H_2_O content), this approach is less reliable.^300^

As in the bulk phase, finite-size effects may also be present when computing diffusivities under confinement. Considering *z* as the confinement direction, Simonnin et al.^359^ have shown that for a LJ fluid the use of periodic boundary conditions in *x* and *y* directions leads to finite-size effects due to the hydrodynamic interactions between periodic images and the constraint of total momentum conservation. Elongated simulation boxes in *x* and *y* directions (*L*_*x*_ ≈ *L*_*y*_ ≫ *L*_*z*_) should be used to avoid such effects. When this is not an option (it is often computationally expensive), analytical expressions may be applied to correct diffusion coefficients.^249,359^ To the best of our knowledge, there is no investigation available in the literature regarding finite-size effects on CO_2_ diffusion under confinement.

#### 3.2.3 Confinement in Natural Media

**Figure 17:**
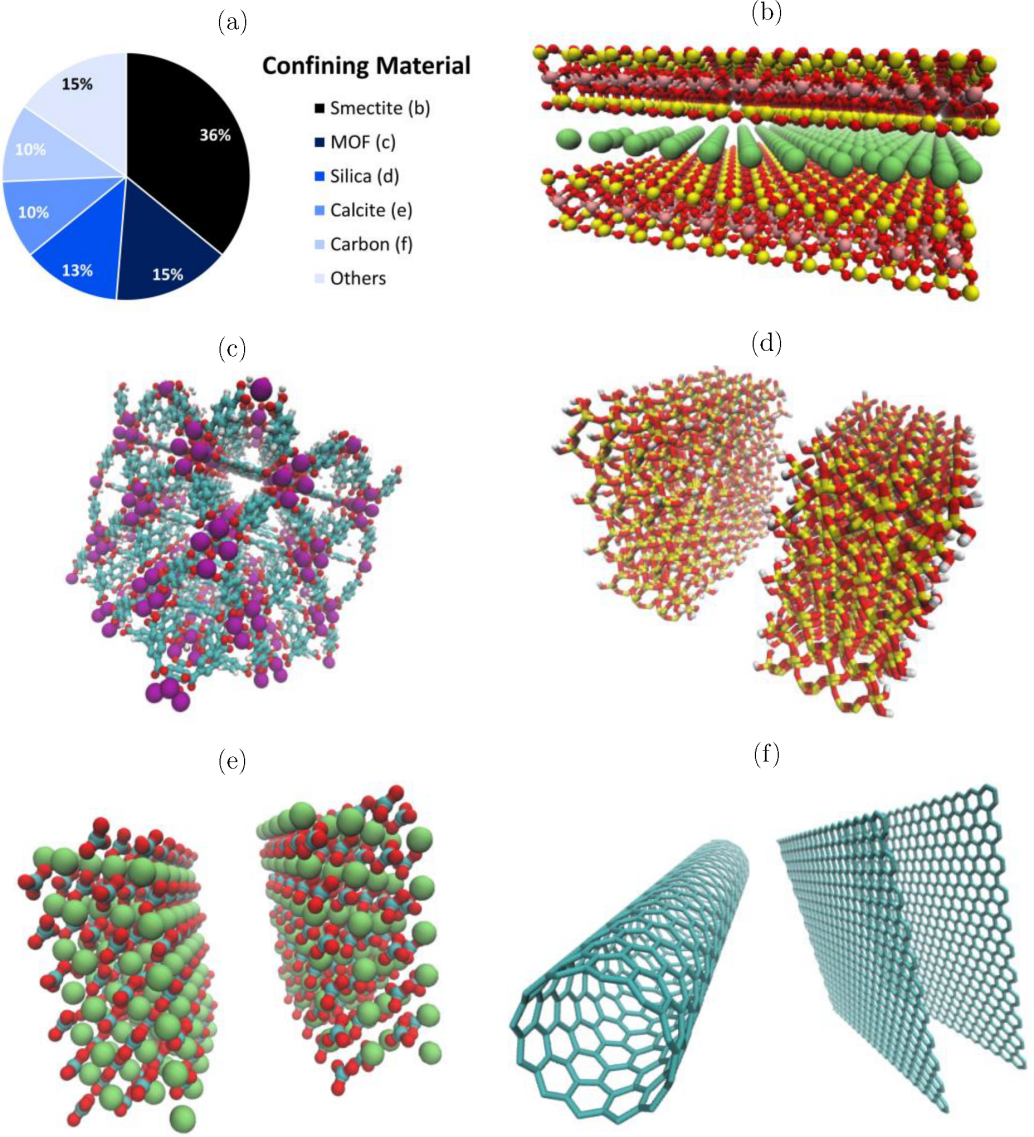
(a) Overview of the relative popularity of confining materials used in molecular simulations in the literature for the investigation of CO_2_–H_2_O diffusion in confinement. An example of the structure of the main confining material is shown: (b) Ca-montmorillonite representing a smectite crystal; (c) UiO-66(Zr) MOF; (d) [001] quartz representing a silica crystal; (e) [1014] calcite crystal; and (f) carbon nanotube and graphene sheets representing carbon materials. The colors red, white, yellow, cyan, green, pink, and purple represent oxygen, hydrogen, silicon, carbon, calcium, aluminum, and zirconium atoms, respectively.

The effect of confinement on CO_2_ depends on the confining material. Figure 17 shows the distribution of confining media used to investigate the CO_2_ diffusion in studies available in the literature.

##### 3.2.3.1 Smectites

Smectites are the most studied material due to their importance in carbon sequestration applications. This clay is a layered aluminosilicate composed of one octahedral (O) sheet with Al as central atom and two adjacent tetrahedral (T) sheets with Si, creating a T–O–T structure. Some of these central atoms are substituted by divalent metals. This creates a partial negative charge in the structure that is balanced by positive counterions located in the interlayer region between two T–O–T structures.^323^ Because of the high hydration energy of counterions, smectites may swell to accommodate H_2_O molecules in the interlayer. It has been experimentally observed that hydrated smectites may also swell in contact with CO_2_, depending on the initial confined H_2_O concentration.^360^ The confinement effect in these conditions is significant, and the molecules distribute themselves in one or two layers.^321^

The different types of smectite can be classified depending on the main substitution of metal atoms and its location.^302^ Montmorillonite (MMT) is the most common smectite and also the most investigated one in regards to CO_2_ diffusion.^264,265,299,301,302,323,325,327,361^ CO_2_ diffusion has also been investigated in the interlayer of hectorite (HEC)^275,326,330^ and beidellite (BEI).^302^

The basal *d*-spacing in the interlayer depends on its relative humidity.^301,323^ For a monolayer (1W), a bilayer (2W), and three layer (3W) H_2_O arrangement, the basal *d*-spacing is expected to be around 12, 15, and 18.5 Å, respectively.^362^ Care should be taken when defining the basal *d*-spacing in MD simulations because not all hydrate states are stable for all clays.^361^ By predefining the basal *d*-spacing, the final equilibrated composition may not correspond to a thermodynamically stable state.^264^ From MD and MC simulations, the stability of the clay can be analyzed through the swelling free energy.^299,323^

Swelling may also occur due to the intercalation of CO_2_ molecules within interlayers.^323,330^ At low CO_2_ concentration and low hydration state, CO_2_ molecules organize themselves parallel to the surface.^276,325,330^ By increasing the H_2_O concentration, CO_2_ adopts other orientations, with some of them pointing perpendicular to the surface.^322,330^ Swelling is not always expected to happen due to CO_2_ intercalation. No evidence of swelling is observed in the presence of CO_2_ for 1W Na-HEC.^275^ The effect of swelling increases CO_2_ diffusivity in the interlayers of smectites.^301,322−324,327^ Transition from 1W to 2W hydration state increases both CO_2_ and H_2_O mobility. The increment is more pronounced on CO_2_ diffusivity because molecules are no longer trapped in a single preferential orientation.^324^ At the same hydration state, H_2_O mobility is higher at lower concentrations of CO_2_ due to the hindering caused by the latter.^327,361^ Kadoura et al.^264^ showed that for a fixed basal *d*-spacing, CO_2_ diffusivity decreases with loading of both CO_2_ and H_2_O due to steric hindrance, but does not depend significantly on the loading of CH_4_. Both CO_2_ and H_2_O molecules simultaneously adsorb in the clay surface and occupy the center region of the interlayer, whereas CH_4_ does not present preferential adsorption. Therefore, the effect of both H_2_O and CO_2_ loading on the CH_4_ diffusion is more pronounced than the effect of CH_4_ loading on CO_2_ diffusion.^264^

The ions in the interlayer reduce the diffusivity of both H_2_O and CO_2_.^263,363^ Severe confinement at 1W structure decreases the mobility of ions the most due to the strong electrostatic interactions with the mineral wall (the diffusion coefficient can be up to 4 orders of magnitude lower than the bulk).^301^ Different cations may occupy the interlayer space to balance the surface charge. By fixing an ion-independent basal *d*-spacing, Kadoura et al.^301^ have concluded that the diffusion of CO_2_ is mostly independent of the cation type. Cations with different hydration energies could lead to different hydration and swelling of the clay, which may affect the diffusion of CO_2_.^324^ The residence time between CO_2_ and ions is short, and the activation energy for H_2_O molecules to move out of the first coordination shell of ions is 5 times larger than the activation energy for CO_2_.^325,330^ The CO_2_–ion interaction is weak compared to their respective interaction with H_2_O molecules. Due to the repulsions, CO_2_ may change the clay wettability.^324^ In the presence of CO_2_, ion migration to the clay basal surface may screen part of the surface charge, increasing the surface hydrophobicity.^323^

Zhang et al.^327^ have performed a compression test in MMT intercalated with CO_2_ and H_2_O by deforming the cell parameters. The self-diffusion coefficient of both species decreases drastically with compression loading and approaches zero at the end of the test. The mineral stiffness is increased by the process of intercalation of both CO_2_ and H_2_O.^327^

Owusu et al.^265^ have investigated the diffusion of different gases (CO_2_ included) in H_2_O confined by MMT. By increasing the pore size, CO_2_ diffusion coefficient converges asymptotically to CO_2_ unconfined diffusion. The diffusion is inversely proportional to the hydrodynamic radius of the gas. The authors have investigated the temperature influence on diffusion. As expected, by increasing temperature, the mobility of both CO_2_ and H_2_O increases. The diffusion activation energy is changed by the confinement: for polyatomic molecules such as CO_2_ and CH_4_, the activation energy is higher than in the bulk H_2_O,^265^ which means that CO_2_ diffusion is less dependent on temperature under confinement.

**Figure 18:**
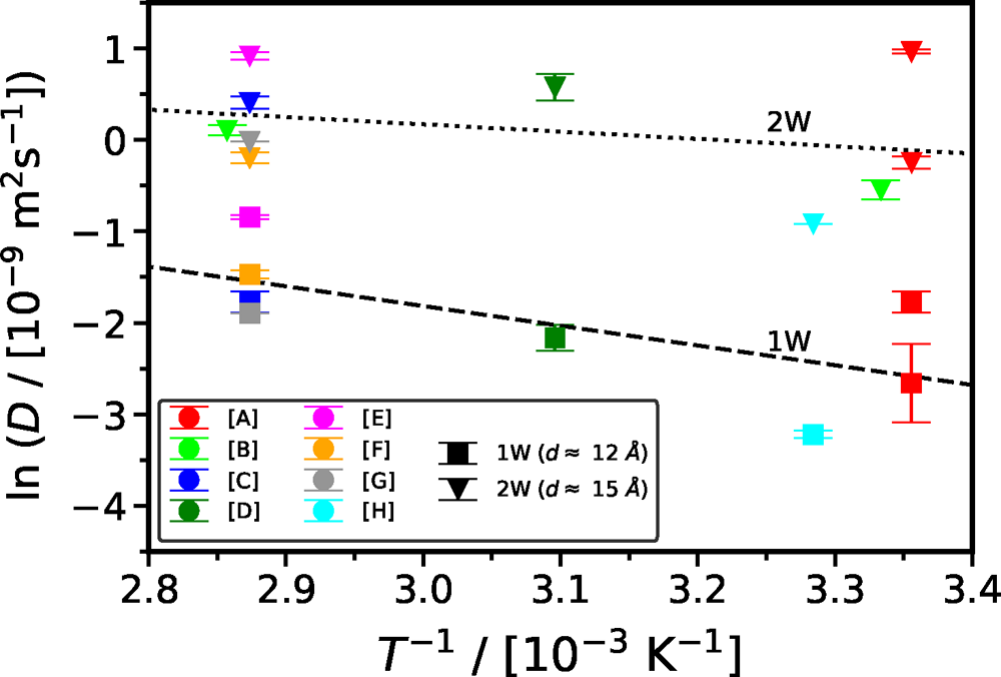
CO_2_ self-diffusion coefficient in Na-MMT at different temperatures for the 1W (square symbols) and 2W (triangle symbols) hydration states. The black dashed and dotted lines represent linear interpolation of Arrhenius equation for the 1W and 2W states, which are given by ln *D*/*D*_0_ = −2157.2/T + 4.7 and ln *D*/*D*_0_ = −798.1/T + 2.6, respectively, where *D*_0_ = 10–9 m2 s–1. Legend: [A] Kadoura et al.;^264^ [B] Owusu et al.;^265^ [C] Botan et al.;^299^ [D] Kadoura et al.;^301^ [E] Makaremi et al.;^302^ [F] Myshakin et al.;^323^ [G] Rahromostaqim and Sahimi;^324^ and [H] Zhang et al.^327^

Figure 18 shows a compilation of the results reported for CO_2_ diffusion coefficient in the interlayer of MMT.  is plotted as a function of 1/*T* to verify the correspondence to Arrhenius equation (i.e., ). A wide range of diffusivities is obtained for similar temperatures and hydration states. The main factors that may cause this dispersion are the fluid composition and density, the force field selection, the definition of the basal distance, and the method of computing the diffusion coefficient. At the same temperature and hydration state (2W), Kadoura et al.^264^ have obtained diffusion coefficients different from each other by a factor of 3. The lower the H_2_O concentration (400 compared to 600 kg m^–3^), the higher the diffusivity. The number of H_2_O and CO_2_ molecules should be defined in GCMC simulations before the MD simulation, to avoid simulation artifacts caused by an arbitrary choice of the number of particles. The usual basal *d*-space definition is the pore distance plus half the width of each T–O–T structure. Owusu et al.^265^ have considered only the pore distance, which could cause some disparity when compared to other works if no correction is made. Finally, if the perpendicular component is accounted for in the trace of the diffusion coefficient,^327^ then lower values are obtained compared with the parallel-only diffusion coefficients. By linear interpolation of the  vs 1/*T* plot, the activation energy (*E*_a_) of CO_2_ diffusion in the 1W and 2W hydration states are 17.9 and 6.6 kJ mol^–1^, respectively. The activation energy computed by Owusu et al.^265^ for CO_2_ diffusion in MMT is ca. 11.1 kJ mol^–1^ (no difference caused by the pore size was accounted for).

##### 3.2.3.2 Calcite

Despite the abundance of carbonate-bearing subsurface formations, only a few works have investigated the diffusion of CO_2_ confined by calcite.^263,317,318,322,334^ CO_2_ solubility is reduced by the hydrophilic surface of calcite and the presence of salts, such as NaCl, may further reduce it.^263^ H_2_O at low concentrations increases CO_2_ diffusion by displacing CO_2_ toward the center of the pore due to H_2_O preferential adsorption.^333,334^ Increasing the concentration of both components, the species mobility decreases due to steric hindrance and molecular collisions.^322,333^ For CO_2_ confined between parallel calcite minerals, an anisotropy in the CO_2_ parallel diffusion coefficients is observed due to the calcite plane morphology.^317^ The same anisotropy is also observed in the CO_2_–H_2_O mixture.^333,334^

##### 3.2.3.3 Silica

CO_2_ diffusion has been also investigated in silica nanopores.^328,334,340,354^ The mobility of CO_2_ increases in regions with larger pores. For this reason, CO_2_ diffusion is higher in sepiolite channels than in palygorskite,^328^ and larger in mesopores than micropores of the SBA-15 structure.^340^ Molecules located close to the porous medium surface have low mobility. The displacement of CO_2_ molecules caused by low concentrations of H_2_O in hydrophilic surfaces increase CO_2_ diffusivity.^334,340^ Under severe confinement (6 Å), CO_2_ diffusion coefficient is five times higher in hydrophobic silica than in the hydrophilic silica because of the lower H_2_O content.^354^

##### 3.2.3.4 Other Materials

Others confining materials include kerogen,^332^ kaolinite,^262^ forsterite,^331^ Illite,^324^ and zeolites.^364^ As with the materials discussed earlier, the diffusivity of CO_2_ increases with temperature in kerogen. In the presence of H_2_O, adsorption of CO_2_ onto functional groups of kerogen is reduced.^332^ The hydrophobic surfaces of kaolinite promote a slightly higher parallel diffusion of CO_2_ than the hydrophilic surfaces for pressures up to 35 MPa.^262^ Rahromostaqim and Sahimi^324^ have investigated CO_2_–H_2_O diffusion confined by mixed layers of MMT and Illite, a mica mineral. They showed that the swelling and ion hydration depends on the charge location of the mineral. Within the bilayer space, the diffusivities of both CO_2_ and H_2_O increase with the H_2_O-to-CO_2_ ratio.^324^ Kerisit et al.^331^ have studied the behavior of CO_2_–H_2_O in the interface of a forsterite mineral. A phase separation occurs, and a H_2_O film forms at this mineral surface. The diffusivity of CO_2_ and H_2_O are similar in both aqueous and CO_2_-rich phase. In the transition interface region, CO_2_ is less hydrated by other H_2_O molecules compared to their hydration in the bulk region, which results in a higher CO_2_ diffusivity than H_2_O diffusivity in this region.^331^ Wang et al.^364^ have investigated the diffusion of flue gas (CO_2_, NO, NO_2_ N_2_, O_2_, SO_2_ and H_2_O) in zeolites (13X and 5A). The authors reported a correlation between the guest molecule size and its diffusivity, with triatomic molecules obtaining a lower diffusion coefficient. Due to the strong binding force between water molecules and the zeolite framework, no detectable H_2_O diffusion was obtained with reasonable accuracy. As expected, the higher the temperature or the pore sizes (zeolite 13X), the higher the mobility and the diffusion coefficient of all molecules.^364^

#### 3.2.4 Confinement in Artificial Media

##### 3.2.4.1 Metal–Organic Frameworks

MOFs are crystal-like structures composed of metal clusters and organic linkers. Due to their potential to separate CO_2_ from flue gas, the diffusion of CO_2_ in various MOFs at different conditions have been investigated.^344−347^ The diffusion behavior of CO_2_ in this confining medium depends on the crystal structure and the loading.

Diffusion and adsorption show opposite trends, i.e., the species with higher adsorption energy tend to have lower mobility. Mera et al.^347^ investigated the diffusion of the CO_2_–N_2_–H_2_O mixture in three MOFs (IRMOF-1, Cu-BTC, and MIL-47). Although Cu-BTC has the narrowest pores, the reduction in pure CO_2_ diffusion is higher in the confinement imposed by MIL-47 due to the stronger interactions between the adsorbate and the framework. In the presence of H_2_O, CO_2_ diffusion coefficient in MIL-47 is increased by 1 order of magnitude. The competition between CO_2_ and H_2_O for the active sites increases the mobility of both species. The opposite occurs in the mixture diffusion in Cu-BTC, in which the species have a lower diffusivity compared to its pure components diffusion.^347^

Magnin et al.^345^ investigated the CO_2_ diffusion in UiO-66 at different loadings of CO_2_ and H_2_O. At lower pressures (lower loadings), CO_2_ preferentially adsorbs in the tetrahedral cages and the diffusion mechanism is mainly cage hopping.^345,346^ By increasing CO_2_ loading, its mobility is reduced due to the increase of CO_2_–CO_2_ collisions and reduction in the MOF free volume. In a different MOF, CALF-20, further increment in CO_2_ loading could actually increase CO_2_ diffusivity because of the presence of more than one CO_2_ per cage could make their interaction with the solid surface weaker.^346^

In UiO-66 MOF, H_2_O acts as an extra sorbent medium for CO_2_ diffusion. The tortuosity created by the H_2_O network, the CO_2_–H_2_O attractive interactions, and the occupied pore volume at high H_2_O loading are some of the reasons for the reduction in CO_2_ mobility in the presence of H_2_O.^345^ In CALF-20, the enthalpies of adsorption of CO_2_ and H_2_O have similar magnitudes, which results in similar values for their diffusion coefficients.^346^ On the other hand, in the Mg-MOF-74, where the adsorption energy between water and the open metal sites is stronger, CO_2_ diffusion coefficient can be an order of magnitude higher than H_2_O.^344^

Darcy’s law describing the fluid flow in the porous media, fails to predict the fluid transport in nanopores by neglecting the adsorption. Magnin et al.^345^ have computed the permeance, which corrects Darcy’s law, using the confined fluid diffusivity. From the nano-Darcy expression, they show that the macroscopic fluid flow in UiO-66 MOF decreases with H_2_O loading, following the behavior predicted by the diffusion mechanisms.^345^

**Figure 19:**
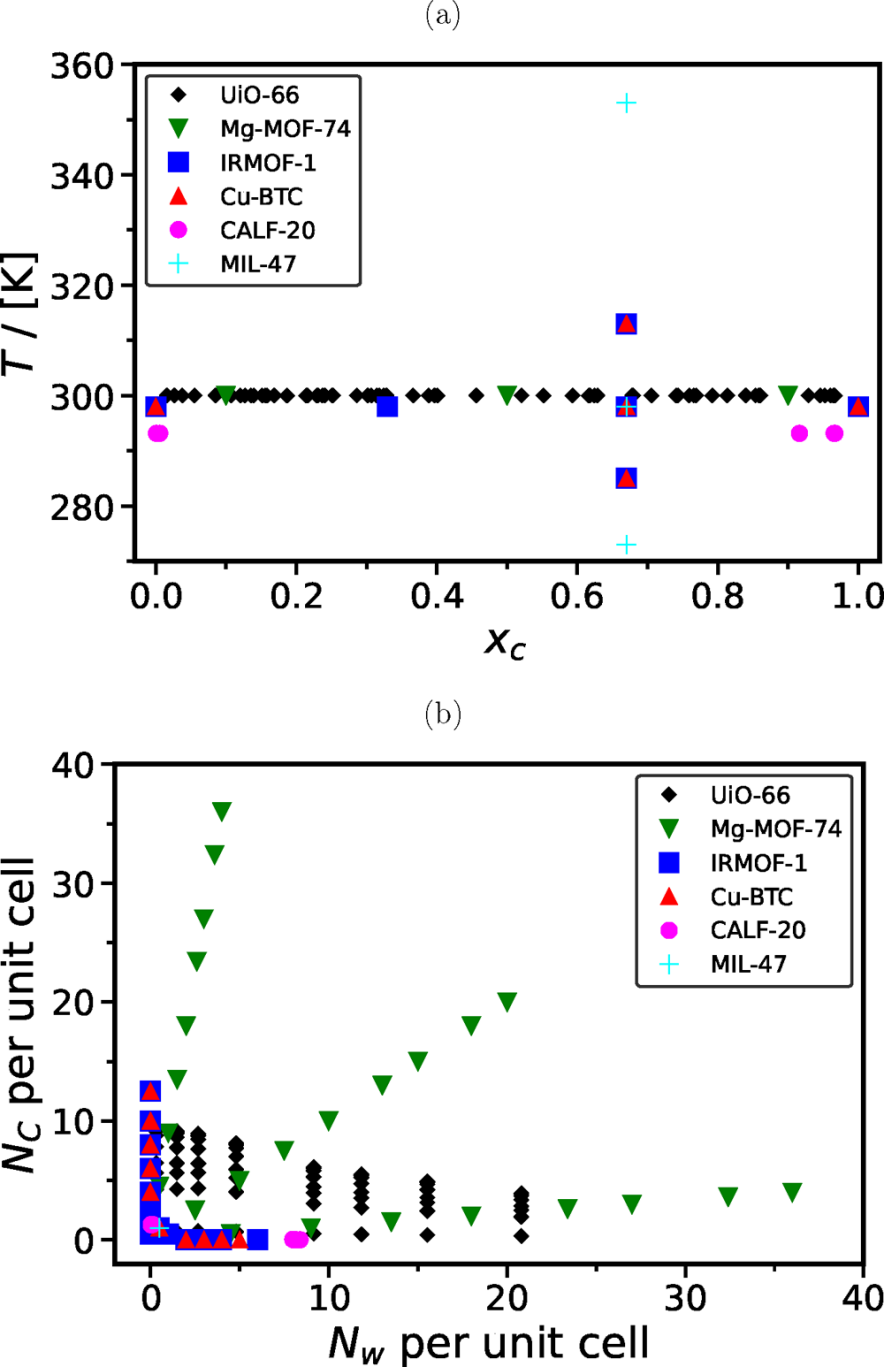
Conditions with available data in the literature for CO_2_ diffusion confined in metal organic frameworks UiO-66,^345^ IRMOF-1,^347^ Cu-BTC,^347^ MIL-47,^347^ Mg-MOF-74,^344^ and CALF-20.^346^ (a) Temperature as a function of CO_2_ composition (*x*_CO___2__ = *N*_CO___2__/(*N*_CO___2__+*N*_H___2___O_)), and (b) loading as a function of the number of CO_2_ and H_2_O per unit cell.

Figure 19 shows at which conditions the CO_2_ diffusion in MOFs has been investigated by the studies available in literature. The CO_2_ mole fraction here accounts only for the presence of CO_2_ and H_2_O as guest molecules (not accounting for the N_2_ in the work of Mera et al.,^347^ for instance). The focus so far has been mainly on temperatures ca. 300 K and low loadings (low pressure), with few exceptions. In the future, it could be interesting to further investigate the temperature and pressure effects, because by changing these conditions one can control the adsorption/release of guest molecules in gas capture applications.

##### 3.2.4.2 Carbon Materials

Carbon nanotubes (CNTs) allow for faster CO_2_ diffusion compared to other nanoporous materials.^336^ The larger the pore, the higher the CO_2_ mobility.^300,336,339^ Contrary to other materials, CO_2_ diffusion coefficients in CNTs is almost space-independent. Svoboda et al.^336^ attribute the abnormal higher diffusivity close to the wall to the CO_2_ parallel orientation to the nanotube. The effect of H_2_O on CO_2_ diffusion is a balance between CO_2_ displacement and CO_2_–H_2_O interactions.^300,336^ Because these interactions are stronger than CH_4_–H_2_O interactions, the effect of preadsorbed H_2_O is less pronounced on CO_2_ diffusion than on methane diffusion.^300^ In hydrophobic carbon mesoporous surfaces, such as CMK-5, at high pressures (high loading) the CO_2_ diffusivity is decreased in the presence of H_2_O due to the reduction in the pore free volume.^340^ In most cases, the mobility of species increases with temperature. Zhao et al.^339^ discovered, however, that the diffusion coefficient of hydrogen decreases with temperature in the mixture of CO_2_–H_2_–H_2_O confined by graphene sheets. The increment in the thermal motion of CO_2_ and H_2_O molecules with temperature acts like an extra obstruction to small H_2_ molecules.^339^

## 4 Outlook

In light of the discussion we provided in this review and the currently available data (experimental and MD) on the diffusivity of CO_2_ in H_2_O, we propose several promising directions for future research for both cases of diffusivity in bulk or under confinement.

### 4.1 CO_2_–H_2_O Diffusion in Bulk

• The effect of pressure on the diffusion of CO_2_ in brines needs to be further investigated via: (i) additional experimental measurements, and/or (ii) extensive MD simulations;
• Additional experimental measurements for CO_2_ diffusion in brines are required to provide adequate data for the development of accurate correlations. Emphasis should be given to aqueous salt-solutions (other than NaCl solutions), as well as to geologic formation brines;
• In addition to useful engineering-type correlations of the experimental data, there is a need for the development of theoretically based models for the diffusivity of CO_2_ in pure H_2_O and brines;
• A call for closer collaboration between experimental and simulation groups is stressed to rigorously validate simulation results, thereby deepening the insights into CO_2_ diffusion in H_2_O. Currently in literature, for many systems the experimental data are insufficient for validating the results from MD studies, especially at high temperatures and pressures. An enhanced synergy between experiments and simulations can pave the way for more accurate simulations of CO_2_ in H_2_O;
• Polarizable force fields may offer the potential for a more precise representation of electrostatic interactions in aqueous solutions of CO_2_, increasing the accuracy of MD simulations.^216−218,365,366^ Simulations are needed toward this direction since no data exist for the diffusivity of CO_2_ in H_2_O using polarizable force fields;
• Ab initio MD (AIMD) simulation is another possible method to study the diffusivity of CO_2_ in H_2_O, yielding a more comprehensive understanding of electronic structure in the solution.^367^ Although AIMD simulations have already been used to investigate the reaction mechanism and dynamics of CO_2_ in different solvents,^367−372^ their applicability to computing transport properties is largely hindered by the significant additional computational cost, compared to classical MD, that does not allow for accessing the time scale required to capture the diffusive regime. Nevertheless, with the ever-increasing computational power being available, AIMD could be an interesting route to explore further;
• Introducing machine learning techniques into force field parametrization is possible to increase the predictive accuracy by discerning patterns in extensive data sets.^373^ This field is already very active, nevertheless, more efforts can focus on the CO_2_–H_2_O system;
• Currently, the behavior of CO_2_ diffusivity at near-critical H_2_O is not well understood. Additional MD simulations are required to produce the necessary data at these conditions. An advancement in this area will facilitate the refinement of the engineering-type correlations, and thus, allow for the development of more accurate predictive tools.

### 4.2 CO_2_–H_2_O Diffusion under Confinement

• Experimental investigation of the mixture diffusion mechanisms with techniques such as QENS and NMR would be a powerful route to explore;
• When performing MD simulations, care should be taken to choose the initial configuration and the method. We advocate the use of GCMC to determine the composition and loading for a giving state, and the use of methods that account for the nonhomogeneity of the confined fluid to compute the diffusion coefficients;
• From a methodological perspective, the study of transport diffusion coefficients using NEMD simulations to account for the collective transport and the investigation of possible finite-size effects in the confined CO_2_ diffusion is an interesting future directive;
• Diffusion within confining materials, such as smectites, have been extensively studied. Others, however, such as calcite, which is a mineral abundant in subsurface formations, needs further investigation since it is important for many applications, e.g., CCS.

## 5 Conclusions

In this review paper, experimental data for the diffusion coefficient of CO_2_ in pure H_2_O are collected and discussed in detail. The experimental data are used to develop simple and computationally efficient correlations. These correlations are applicable to temperatures from 273 K and 0.1 MPa to 473 K and pressures up to 45 MPa. At this pressure and temperature range, the diffusion coefficient of CO_2_ in H_2_O has a very weak dependence on pressure. Therefore, the proposed correlations are only temperature-dependent. The proposed correlations could be useful for engineering calculations that are related to a number of industrial and environmental processes. Finally, experimental data for the diffusion coefficient of CO_2_ in brines are collected and their dependency on temperature, pressure and salinity have been thoroughly examined and reported.

Along with the experimental data, in this review, a detailed discussion on the available MD studies of CO_2_ diffusivity in aqueous solutions is provided. The focus is on the force field combinations, the data for diffusivities at low and high pressures, the finite-size effects, and the correlations using MD data. The vast majority of the available MD studies of CO_2_ diffusivity in H_2_O report data at the infinite dilution limit (i.e., 1 to 5 solute molecules). The very few data available for higher CO_2_ compositions are also provided and useful analysis is performed. A short discussion related to CO_2_ diffusivity in carbonated hydroalcoholic drinks is also available.

For certain applications, e.g., CCS, a confining structure can constrain the CO_2_ mobility, and consequently reduce CO_2_ diffusion coefficients. Here, the main methods to compute the diffusivity of confined CO_2_ are reviewed and the main natural and artificial confining media (i.e., smectites, calcites, silica, MOFs, and carbon materials), focusing primarily on MD simulations and secondarily on experimental studies are discussed. Smectites were found to be the most studied material due to their swelling, which generates an interlayer space capable of intercalating CO_2_ and H_2_O. The diffusion of CO_2_ and H_2_O under confinement is driven by a balance between adsorption and steric hindrance. For hydrophilic surfaces, water at lower concentrations increases CO_2_ mobility due to preferential adsorption of H_2_O. Based on the analysis and discussion, an outlook containing possible, useful, future research paths for advancing the field of CO_2_ diffusivity in H_2_O at the bulk phase and in confinement is devised.

## Supporting Information Available

Raw experimental and simulation data shown in the figures, along with the available statistical uncertainties (XLSX)
